# Clean Synthesis of *N*‐4‐Alkylamino‐7‐chloroquinoline Cyclic Imides: Influence of Lipophilicity on Antibacterial and Ecotoxicological Properties

**DOI:** 10.1002/cbdv.71688

**Published:** 2026-09-10

**Authors:** Agenor P. Luz‐Filho, Thais C. Silva, Matheus B. Silva, Abraão P. Sousa, Helivaldo D. S. Souza, Priscila S. V. Lima, Alexandre Almeida‐Júnior, Fábio C. Sampaio, Petrônio F. Athayde‐Filho, Gabriela F. Fiss

**Affiliations:** ^1^ Laboratório de Pesquisa em Bioenergia e Síntese Orgânica (LPBS) Department of Chemistry Universidade Federal da Paraíba (UFPB) João Pessoa Brazil; ^2^ Núcleo de Química de Heterociclos (NUQUIMHE) Department of Chemistry Universidade Federal de Santa Maria Santa Maria Brazil; ^3^ Laboratório De Biologia Bucal (LABIAL) Department of Clinical and Community Dentistry UFPB João Pessoa Brazil

**Keywords:** *Artemia salina*, environmental chemistry, lipophilicity, phthalimide, succinimide

## Abstract

Motivated by the promising antimicrobial and ecotoxicological effects of 4‐alkylamino‐7‐chloroquinoline and *N*‐alkylphthalimide derivatives, these privileged nuclei were linked to investigate how lipophilicity influences antibacterial activity and ecotoxicity. For this purpose, seven 4‐alkylamino‐7‐chloroquinolines (**2a–g**) were prepared, of which four were used as precursors (**2a–d**) and reacted with different anhydrides. Using a clean synthetic approach, 4‐alkylamino‐7‐chloroquinoline–succinimides (**3a–d**) and –phthalimides (**5a–d**) were obtained in yields of 60%–99% after only filtration with distilled water. In vitro assays were performed to determine the minimum inhibitory/bactericidal concentration (MIC/MBC) against *Klebsiella pneumoniae*, *Pseudomonas aeruginosa*, *Staphylococcus aureus*, and *Streptococcus mutans*. Lipophilicity (log *P*) and toxicity were predicted using the pkCSM program. In vitro toxicity tests on *Artemia salina* larvae were used as an indicator of ecotoxicity. 4‐Alkylamino‐7‐chloroquinolines (**2b**, **2f**, **2g**) revealed a moderate MIC of 156.25 µg·mL^−1^ against Gram‐(–) and Gram‐(+) bacteria, whose predicted toxicity does not pose a potential risk to human health. Results indicate a correlation between increased lipophilicity and higher ecotoxicity, where less lipophilic compounds were less toxic to *A. salina* (LC_50_ 133.01–777.50 µg·mL^−1^) than 4‐alkylamino‐7‐chloroquinoline–phthalimides, suggesting further investigation of their cytotoxicity.

## Graphical abstract

For series **2a–d**, there appears to be optimal activity against *Klebsiella pneumoniae* when lipophilicity is intermediate (log *P* 2.64 for **2b**). Similarly, for series **2e–g**, activity against *Pseudomonas aeruginosa*, *Staphylococcus aureus* and *Streptococcus mutans* also increases (log *P* 3.71 for **2f**). More lipophilic 4‐alkylamino‐7‐chloroquinolines and their cyclic imide derivatives tend to be more toxic to *Artemia salina* larvae.

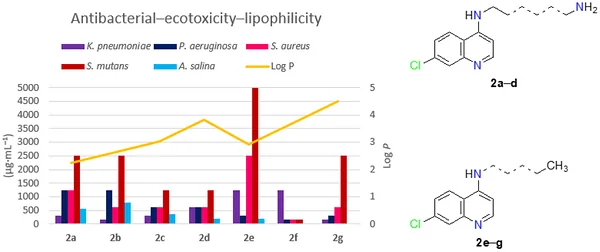

## 1 Introduction

The natural phenomenon of antimicrobial resistance (AMR) results from progressive genetic changes in pathogens over time. However, this process has been intensified by human activity, especially by the inappropriate and excessive use of antimicrobials in the prevention and treatment of infections. The search for novel drug candidates is something constantly researched by the scientific community, given the need to improve commercially available therapeutic agents [1].

Some nuclei are known as privileged due to chemical structures with a high probability of interaction with specific targets. According to the United States Food and Drug Administration (U.S. FDA), nitrogen heterocyclic compounds are present in more than half of approved pharmaceuticals [2]. Among these compounds, the quinoline nucleus, present in drugs such as chloroquine, a 4‐alkylamino‐7‐chloroquinoline, and ciprofloxacin, an antibiotic, deserves special mention due to its synthetic viability and versatility [3]. The relevance of 4‐alkylamino‐7‐chloroquinolines to the scientific community is undeniable, where structure–activity relationship (SAR) studies suggest that the alkylamino and chlorine groups at positions C4 and C7, respectively, are essential for antiplasmodial activity [4, 5]. Furthermore, 4‐alkylamino‐7‐chloroquinolines have been explored for their green [6, 7] and supramolecular [8, 9] chemistry, and antimicrobial properties [7, 10, 11, 12].

**FIGURE 1:**
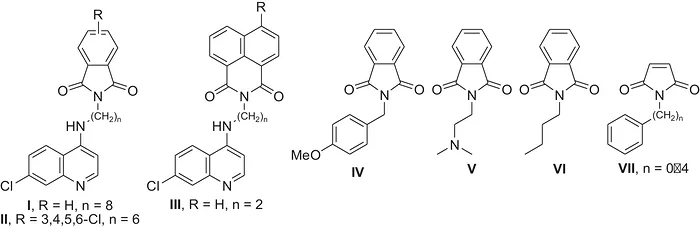
Promising cyclic imide derivatives.

Previous studies have demonstrated growing interest in 4‐alkylamino‐7‐chloroquinolines and their cyclic imide derivatives due to their therapeutic potential. Cyclic imides can be subdivided into glutarimides, maleimides, naphthalimides, phthalimides, succinimides, among others [13]. In particular, phthalimides have been the subject of study regarding their synthetic methods and diverse activities—as covered in review articles [14, 15]—especially concerning antitumor activity [16]. In this context, 4‐alkylamino‐7‐chloroquinoline–phthalimides were evaluated for antitubercular [17] and antiplasmodial [18] activities. The combination of the phthalimide ring with an octyl linker (**I**, Figure 1) proved to be the most active against *Mycobacterium tuberculosis*, but the most cytotoxic on murine macrophage cells [17], while the optimal combination of the tetrabromophthalimide ring with a hexyl linker (**II**, Figure 1) proved to be the most selective between murine macrophage cells and *Plasmodium falciparum* [18]. Among other *N*‐4‐alkylamino‐7‐chloroquinoline cyclic imides, 4‐alkylamino‐7‐chloroquinoline–naphthalimides and glutarimide derivatives were synthesized by the reaction of 4‐alkylamino‐7‐chloroquinolines with 1,8‐naphthalic anhydrides, using *N*‐methyl‐2‐pyrrolidone (NMP) as solvent at 150°C for 2 h. The scaffolds were evaluated for activity against chloroquine‐resistant *P. falciparum* and toxicity toward Vero kidney cells. The unsubstituted hybrid with an ethyl linker (**III**, Figure 1), being less lipophilic, was the least cytotoxic and, therefore, the most selective [19].

The cytotoxicity and antimicrobial effects of *N*‐alkylphthalimides have also been investigated [20, 21]. The most promising antimicrobial results showed that *N*‐(4‐methoxybenzyl)phthalimide (**IV**, Figure 1) was active against Gram‐(+) bacteria *Micrococcus luteus* and *Listeria monocytogenes*, with halos greater than 30 and 20 mm, respectively, and *N*‐(2‐(dimethylamino)ethyl)phthalimide (**V**, Figure 1) being active against *L. monocytogenes*, with a halo greater than 20 mm [20]. More recently, Shaik et al. [21] investigated the antibiofilm and anti‐hyphal effects of a series of *N*‐alkylphthalimides. *N*‐Butylphthalimide (**VI**, Figure 1) was the most potent, inhibiting biofilm formation by *Candida albicans* and *Staphylococcus epidermidis*, with a minimum inhibitory concentration (MIC) of 100 µg·mL^−1^, polymicrobial biofilms of *C. albicans* and *S. epidermidis*, with an MIC of 200 µg·mL^−1^, and hyphae. Some *N*‐alkylmaleimides (**VII**, Figure 1) exhibited activity against bacteria such as *Escherichia coli*, *Klebsiella pneumoniae*, and *Staphylococcus aureus*, among others, and fungi such as *C. albicans*, *Microsporum* canis, and *Penicillium*, among others. The distance between the imide and aromatic rings appears to be a determining factor for antifungal activity, which is enhanced by a longer linker [13].

Variations in the hydrocarbon chain length have been used to modulate lipophilicity and, consequently, the mechanisms of microbial resistance. The partitioning of an organic compound in an octanol:water system is an adequate simulation of the membrane:water interface [22]. Thus, lipophilicity, which has a direct impact on oral absorption, permeability, and toxicity, can be estimated using this partition coefficient, where measuring the concentrations of a substance in both phases results in log *P*_O/W_ (log *C*_O_/*C*_W_), with the ideal range being between 1 and 3 [23].

Just as important as developing potentially active compounds is releasing less toxic bioactives into the environment. Considering the recurring disposal of pharmaceutical waste in aquatic environments, in vitro toxicity tests using *Artemia salina* larvae have been widely employed to evaluate the ecotoxicity of bioactives as a preliminary cytotoxicity indication [24]. In a previous study by our research group [7], it was observed that toxicity to *A. salina* tends to be lower for less lipophilic 4‐alkoxy/alkylamino‐7‐chloroquinolines. Furthermore, our research group has systematically investigated the antimicrobial and ecotoxic effects of 4‐alkoxy/alkylamino‐7‐chloroquinolines [7, 25] and *N*‐alkylphthalimides [26]. Based on the promising results obtained for these privileged nuclei, this work proposes a clean synthesis and an in silico/vitro study of 4‐alkylamino‐7‐chloroquinolines linked to cyclic imides through the alkyl side chain at the 4‐position of the quinoline ring. The main question is the investigation of the antibacterial activity and ecotoxicity of *N*‐4‐alkylamino‐7‐chloroquinoline cyclic imides as a function of lipophilicity.

## 2 Results and Discussion

### 2.1 Chemistry

**SCHEME 1:**
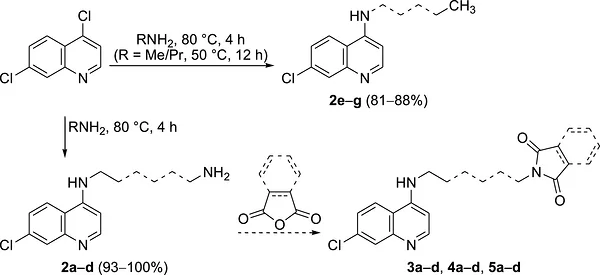
Synthetic design for obtaining *N*‐4‐alkylamino‐7‐chloroquinoline cyclic imides.

The synthetic design for obtaining *N*‐4‐alkylamino‐7‐chloroquinoline cyclic imides is presented in Scheme 1. The proposed synthetic route begins with the preparation of 4‐alkylamino‐7‐chloroquinolines (**2a–g**) from the nucleophilic aromatic substitution (S_N_Ar) reaction of 4,7‐dichloroquinoline with ethan‐1,2‐diamine (**1a**), propan‐1,3‐diamine (**1b**), butan‐1,4‐diamine (**1c**), hexan‐1,6‐diamine (**1d**), methylamine (**1e**), propylamine (**1f**), and pentylamine (**1g**). The precursors **2a–d** were reacted with succinic, maleic, and phthalic anhydrides, with the aim of obtaining 4‐alkylamino‐7‐chloroquinoline–succinimides (**3a–d**), –maleimides (**4a–d**), and –phthalimides (**5a–d**), respectively.

**SCHEME 2:**
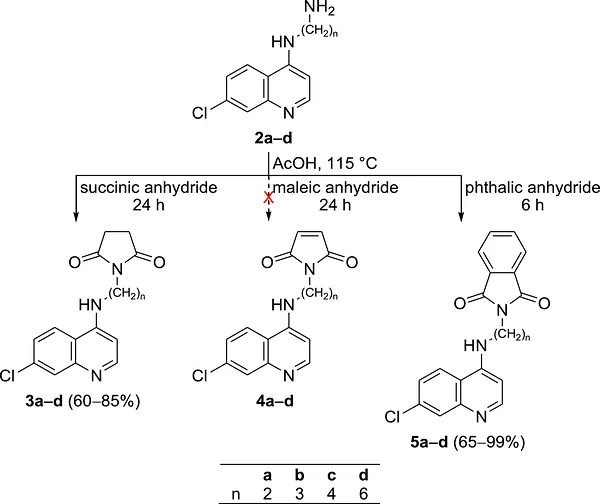
Reagents and reaction conditions for obtaining *N*‐4‐alkylamino‐7‐chloroquinoline cyclic imides.

The compounds **2a–d** were synthesized for use as precursors in the subsequent reaction step, while the compounds **2e–g** were synthesized for bioactivity/lipophilicity comparison purposes. Thus, according to Fiss et al. [7], 4‐alkylamino‐7‐chloroquinolines (**2a–g**) were obtained through a cleaner approach, without the use of solvent and an additional purification step. Next, the precursors **2a–d** were reacted with different anhydrides. Based on a previously described method [17, 18], with some adjustments, the reactions were carried out by conventional heating at 115°C in acetic acid (AcOH) as solvent for 24 h for succinic and maleic anhydrides, and 6 h for phthalic anhydride. 4‐Alkylamino‐7‐chloroquinoline–succinimides (**3a–d**) and –phthalimides (**5a–d**) were obtained in yields of 60%–85% and 65%–99%, respectively. Under the reaction conditions tested, 4‐alkylamino‐7‐chloroquinoline–maleimides (**4a–d**) were not obtained (Scheme 2).

According to Rani et al. [17, 18], 4‐alkylamino‐7‐chloroquinoline–phthalimide **5a** was initially obtained by reacting the precursor **2a** with phthalic anhydride by conventional heating at 110°C in AcOH as solvent for 6 h. The yield of 4‐alkylamino‐7‐chloroquinoline–phthalimide **5a** was improved from 50% to 89% by microwave heating at 160°C for 2 min. Rani et al. [17, 18] tested other solvents besides AcOH, such as acetonitrile, dimethylformamide, dimethylsulfoxide (DMSO), and ethanol. The best solvents for use in conventional and microwave heating were AcOH and DMSO, respectively. Thus, 4‐alkylamino‐7‐chloroquinoline–phthalimides (**5a‐d**) were obtained after two additional liquid–liquid extraction and recrystallization steps with yields ranging from 74% to 89% [17, 18]. Some of these compounds, including ethyl and propyl linkers, had already been synthesized previously, as well as 4‐alkylamino‐7‐chloroquinoline–maleimides, but using a more ingenious synthetic method [27] (Table 1).

**TABLE 1:** Comparative summary of *N*‐4‐alkylamino‐7‐chloroquinoline cyclic imides.

Compd.	Synthetic conditions	Separation step	Yield (%)	Assays		Ref.
				Activity	Toxicity	
**3a**	AcOH, 115°C, 6 h	Precipitation in water	60	Antibacterial	*A. salina* larvae	In this work
**3b**			85			
**3c**			66			
**3d**			67			
**4a**	DCM, 0°C, 2 h; AcONa, AcOAc, 60°C, 5 h	Column chromatography	98	—	—	[27]
**4b**			75			
**4c**	—	—	—	—	—	—
**4d**						
**5a**	DCM, 0°C, 2 h; AcONa, AcOAc, 60°C, 5 h	Column chromatography	90	—	—	[27]
	AcOH, 110°C, 6 h	Liquid–liquid extraction	50	Antitubercular Antiplasmodial	Murine macrophage cells	[17, 18]
	DMSO, MW, 160°C, 2 min		89			
	AcOH, 115°C, 6 h	Precipitation in water	65	Antibacterial	*A. salina* larvae	In this work
**5b**	DCM, 0°C, 2 h; AcONa, AcOAc, 60°C, 5 h	Column chromatography	88	—	—	[27]
	DMSO, MW, 160°C, 2 min	Liquid–liquid extraction	86	Antitubercular Antiplasmodial	Murine macrophage cells	[17, 18]
	AcOH, 115°C, 6 h	Precipitation in water	99	Antibacterial	*A. salina* larvae	In this work
**5c**	DMSO, MW, 160°C, 2 min	Liquid–liquid extraction	74	Antitubercular Antiplasmodial	Murine macrophage cells	[17, 18]
	AcOH, 115°C, 6 h	Precipitation in water	76	Antibacterial	*A. salina* larvae	In this work
**5d**	DMSO, MW, 160°C, 2 min	Liquid–liquid extraction	87	Antitubercular Antiplasmodial	Murine macrophage cells	[17, 18]
	AcOH, 115°C, 6 h	Precipitation in water	90	Antibacterial	*A. salina* larvae	In this work

In the present work, 4‐alkylamino‐7‐chloroquinoline–succinimides (**3a–d**) and –phthalimides (**5a–d**) were obtained in moderate to good yields after filtration with distilled water, without the need for additional purification steps such as liquid–liquid extraction and/or column chromatography, in accordance with the first principle (prevention) of Green Chemistry [28]. As a cleaner synthetic strategy, the solvent choice was a weak organic acid (AcOH), water‐soluble, inherent to soil, and biodegradable. Although AcOH has safety and environmental scores of 3, its health score of 7 is problematic, requiring caution during handling [29]. Regarding the second principle (atom economy) of Green Chemistry [30], the atomic efficiency, calculated by dividing the mass of the desired product by total mass of reactants and multiplying by 100, ranged from 56% (for compound **3a**) to 93% (for compound **5b**), while the Environmental factor (E‐factor), calculated by dividing total mass of waste (including solvents) by the mass of the desired product, ranged from 3.6 to 7.5. Furthermore, these green syntheses are reproducible, and the process is scalable.

**SCHEME 3:**
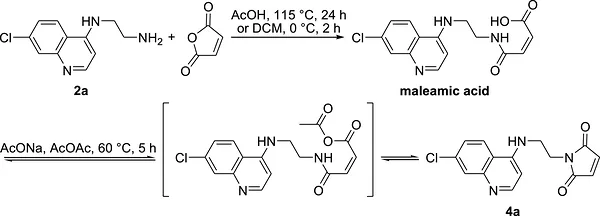
Proposed hypothetical formation of 4‐alkylamino‐7‐chloroquinoline–maleimide **4a**.

For 4‐alkylamino‐7‐chloroquinoline–maleimides (**4a–d**) not obtained using reactional conditions tested in this work (AcOH, 115°C, 24 h) or, according to Rojas and Kouznetsov [27], using dichloromethane (DCM) as solvent at 0°C for 2 h in a first step, maleamic acid is formed. In a second step, according to Rojas and Kouznetsov [27], using 10 mol% sodium acetate (AcONa) in sodium anhydride (AcOAc) as solvent at 60°C for 5 h, the reaction goes through the formation of a non‐isolated intermediate before leading to the formation of 4‐alkylamino‐7‐chloroquinoline–maleimide **4a** (Scheme 3).

Therefore, in this work, maleamic acid may have been formed, but during the treatment step of the reactional mixture, which was washed with a saturated sodium bicarbonate solution, maleamic acid may have been lost in the aqueous phase. This proposal corroborates reports in the literature [13], where attempts to obtain maleimides without isolating maleamic acid proved inefficient, resulting in the production of maleimide derivatives in low yields (∼10%).

The newly synthesized compounds were characterized by spectroscopic techniques of infrared (IR) and ^1^H and ^13^C nuclear magnetic resonance (NMR). Furthermore, high‐resolution mass spectrometry (HRMS) analyses confirmed the exact masses of 4‐alkylamino‐7‐chloroquinoline–succinimides (**3a–d**) with errors of less than 5 ppm. With regard to the ^1^H NMR spectra of 4‐alkylamino‐7‐chloroquinoline‐succinimides (**3a–d**), the main signals that confirm the obtaining of the products are the absence of the signal referring to the NH_2_ group belonging to the precursors, and the presence of a singlet referring to the methylene hydrogens of the succinic ring at 2.60–2.61 ppm. According to the ^13^C NMR spectra, the main set of signals confirming the formation of the products is the signal corresponding to the carbonyl carbon at 177.1–177.8 ppm, and a single signal corresponding to the two methylene carbons of the succinic ring at 27.6–28.1 ppm. Finally, in the IR spectra, the most significant changes compared to their respective precursors were the appearance of stretching related to C═O bonds at 1685–1766 cm^−1^, an increase in stretching related to C─H bonds at 2854–2939 cm^−1^, and a decrease in the intensity of the band related to the N─H bond at 3201–3525 cm^−1^.

### 2.2 Antibacterial Evaluation

The antibacterial activity of 4‐alkylamino‐7‐chloroquinolines (**2a–g**), 4‐alkylamino‐7‐chloroquinoline–succinimides (**3a–d**) and –phthalimides (**5a–d**) was evaluated against Gram‐(–) and Gram‐(+) bacteria, using the broth microdilution method to determine the minimum inhibitory/bactericidal concentration (MIC/MBC). Data from in vitro antibacterial assays are available in Table 2.

**TABLE 2:** Data from in vitro antibacterial assays of 4‐alkylamino‐7‐chloroquinolines (**2a–g**), 4‐alkylamino‐7‐chloroquinoline–Succinimides (**3a–d**), and –Phthalimides (**5a–d**).

Compound		*n*	MIC/MBC^a^			
			Gram‐(–)		Gram‐(+)	
			*K. pneumoniae* ATCC 13883	*P. aeruginosa* ATCC 27853	*S. aureus* ATCC 15656	*S. mutans* ATCC 25175
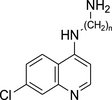	**2a**	2	312.5/625	1250/1250	1250/2500	2500/5000
	**2b**	3	156.25/312.5	1250/1250	625/5000	2500/5000
	**2c**	4	312.5/625	625/1250	625/2500	1250/2500
	**2d**	6	625/1250	625/625	625/1250	1250/2500
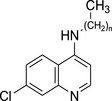	**2e**	0	1250/>5000	312.5/312.5	2500/>2500	>2500/>2500
	**2f**	2	1250/5000	156.25/2500	156.25/2500	156.25/5000
	**2g**	4	156.25/>5000	312.5/>5000	625/5000	2500/>5000
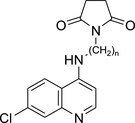	**3a**	2	>5000	>5000	>5000	>5000
	**3b**	3	>5000	>5000	>5000	>5000
	**3c**	4	>5000	>5000	>5000	>5000
	**3d**	6	5000/>5000	>5000	5000/5000	>5000
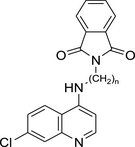	**5a**	2	>5000	>5000	>5000	>5000
	**5b**	3	>5000	>5000	>5000	>5000
	**5c**	4	2500/>5000	>5000	>5000	>5000
	**5d**	6	>5000	>5000	>5000	>5000
Ciprofloxacin	NA		1.95/15.62	15.62/31.25	0.48/0.48	0.24/0.96
Ciprofloxacin hydrochloride			7.80/62.5	15.62/15.62	1.95/1.95	1.95/3.90

^a^ µg·mL^−1^. NA: not applicable.

According to the World Health Organization (WHO) [31], the Bacterial Priority Pathogens List (BPPL) has been compiled by prioritizing pathogenic bacteria based on their level of threat due to antibiotic resistance. The 2024 update categorizes these pathogens into critical, high, and medium priority groups. *K. pneumoniae*, resistant to carbapenems and third‐generation cephalosporins, belongs to the critical group. Carbapenem‐resistant *Pseudomonas aeruginosa* and methicillin‐resistant *S. aureus* (MRSA) are in the high group. The morbidity, mortality, and healthcare costs associated with MRSA cannot be underestimated and remain a major concern due to its persistent prevalence and potentially serious infections. To address the challenges posed by MRSA, a comprehensive approach is needed that combines continuous investment in R&D, enhanced infection prevention and control, global governance and surveillance programs [32].

A growing number of animal experiments and clinical findings have shown that *Streptococcus mutans*, the main pathogenic bacterium causing tooth decay, is not limited to damaging dental tissues. The characteristics of this pathogen may allow *S. mutans* to adhere not only to the surface of teeth, but also to other tissues, such as the vascular endothelium, triggering inflammatory reactions, and causing damage to various organs, thus leading to the emergence of systemic diseases such as cerebral hemorrhage, inflammatory bowel disease, tumors, and infective endocarditis [33].

According to Table 2, 4‐alkylamino‐7‐chloroquinolines **2a–c** and **2e–g** showed moderate antibacterial activity (MIC 156.25–312.5 µg·mL^−1^). Compound **2f** stands out for having a MIC of 156.25 µg·mL^−1^ against strains of *P. aeruginosa*, *S. aureus*, and *S. mutans*, representing the largest antibacterial window in the series. In an analysis by strain type, the most promising results were for *K. pneumoniae* and *P. aeruginosa* strains, which presented a broader set of active compounds.

Despite the moderate antibacterial results for 4‐alkylamino‐7‐chloroquinolines, the activity was not preserved in the final products (**3a–d** and **5a–d**), indicating that 4‐alkylamino‐7‐chloroquinolines linked to succinimide or phthalimide nuclei do not constitute an effective strategy to increase antibacterial activity against the evaluated strains. This behavior suggests that 4‐alkylamino‐7‐chloroquinoline–succinimides (**3a–d**) and –phthalimides (**5a–d**) may have impaired the interaction with the desired targets, either by hindering penetration into the bacterial cell or by reducing affinity with the site of action [34].

In a previous work by our research group [7], when 4‐alkylamino‐7‐chloroquinolines were tested by the inhibition zone method, the best results against *P. aeruginosa* (NEWP 0027) were for compounds **2a–c**, with halos larger than 20 mm. In another study [10], compound **2a** showed activity against *E. coli* (MTCC 119) and *S. aureus* (MTCC 96), inhibiting growth by 79% and 85%, respectively.

Considering the effect of lipophilicity on antibacterial activity, for series **2a–d**, there appears to be optimal activity against *K. pneumoniae* when lipophilicity is intermediate (log *p* = 2.64 for compound **2b**). When lipophilicity is lower (log *p* = 2.25 for compound **2a**) or higher (log *P* 3.03–3.81 for compounds **2c–d**), antibacterial activity decreases. Similarly, for series **2e–g**, activity against *P. aeruginosa*, *S. aureus*, and *S. mutans* increases when lipophilicity is intermediate (log *p* = 3.71 for compound **2f**).

### 2.3 Toxicity Evaluation

Using the pkCSM program [35], log *P* and toxicity parameters were calculated. Additionally, according to an adapted method reported by Fiss et al. [7], in vitro toxicity test of 4‐alkylamino‐7‐chloroquinolines (**2a–g**), 4‐alkylamino‐7‐chloroquinoline–succinimides (**3a–d**) and –phthalimides (**5a–d**) were performed on *A. salina* larvae. A saline solution, in which no nauplii died, and a saline solution containing 3% DMSO and 2% Tween 80, in which the number of dead nauplii was 0.33, were used as negative controls. A potassium dichromate solution was used as a positive control in which all nauplii died. In silico lipophilicity and in silico/vitro toxicity data are summarized in Tables 3 and 4.

**TABLE 3:** In silico lipophilicity and in silico/vitro toxicity data for 4‐alkylamino‐7‐chloroquinolines (**2a–g**), 4‐alkylamino‐7‐chloroquinoline–Succinimides (**3a–d**), and –Phthalimides (**5a–d**).

Compound		*n*	In silico					In vitro
			Log *P*	Toxicity				
				AMES^a^	Hepato	Skin sensitization	*Minnow* Log LC_50_	*A. salina* LC_50_ 24 h
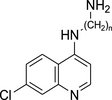	**2a**	2	2.25	No	No	No	1.85	573.09 [7]
	**2b**	3	2.64	No	No	No	1.52	777.50 [7]
	**2c**	4	3.03	No	Yes	No	1.19	368.12 [7]
	**2d**	6	3.81	No	Yes	No	0.53	189.60
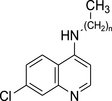	**2e**	0	2.92	No	No	No	1.54	203.87 [7]
	**2f**	2	3.71	No	No	No	1.05	<< 100 [7]
	**2g**	4	4.49	No	No	No	0.39	<< 100 [7]
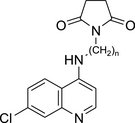	**3a**	2	2.44	Yes	Yes	No	1.80	362.10
	**3b**	3	2.83	Yes	Yes	No	1.47	707.31
	**3c**	4	3.22	No	Yes	No	1.14	133.01
	**3d**	6	4.00	No	Yes	No	0.48	<< 100
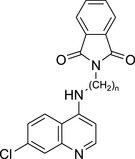	**5a**	2	3.59	No	Yes	No	0.91	<< 100
	**5b**	3	3.98	No	Yes	No	0.58	<< 100
	**5c**	4	4.37	No	Yes	No	0.25	<< 100
	**5d**	6	5.15	No	Yes	No	−0.40	<< 100
Ciprofloxacin hydrochloride	NA		2.00	Yes	Yes	No	1.81	185.68

^a^ Mutagenic potential using bacteria. NA: not applicable. LC_50_: lethal concentration where a substance causes the death of 50% of organisms (µg·mL^−1^). Reference for log LC_50_ on flathead minnows: high acute toxicity < −0.3 [35]. Reference for LC_50_ on *A. salina* larvae after 24 h: highly toxic < 100 ≤ moderately toxic < 500 ≤ slightly toxic < 1000 ≤ nontoxic [36].

**TABLE 4:** Average percentages ± standard deviation (%) of alive *A. salina* nauplii at different concentrations of 4‐alkylamino‐7‐chloroquinolines (**2a–g**), 4‐alkylamino‐7‐chloroquinoline–Succinimides (**3a–d**) and –Phthalimides (**5a–d**), and ciprofloxacin hydrochloride after 24 h.

Compound	Concentration series (µg·mL^−1^)***		
	125	250	375
**2a**	75.84 ± 5.97	65.50 ± 5.97	62.05 ± 0.00
**2b**	95.38 ± 4.00	82.73 ± 0.00	68.94 ± 5.97
**2c**	91.93 ± 8.69	68.94 ± 5.97	51.71 ± 0.00
**2d**	72.39 ± 0.0	24.13 ± 11.94	3.45 ± 5.97
**2e**	68.94 ± 5.97	44.82 ± 5.97	13.79 ± 5.97
**2f**	6.89 ± 5.97	3.44 ± 5.97	0.00 ± 0.00
**2g**	3.44 ± 5.97	0.00 ± 0.00	0.00 ± 0.00
**3a**	95.38 ± 4.00	62.05 ± 10.34	51.71 ± 10.34
**3b**	75.84 ± 5.97	72.39 ± 0.00	68.94 ± 5.97
**3c**	55.16 ± 11.94	17.23 ± 11.94	0.00 ± 0.00
**3d**	0.0 ± 0.00	0.00 ± 0.00	0.00 ± 0.00
**4a**	0.0 ± 0.00	0.00 ± 0.00	0.00 ± 0.00
**4b**	0.0 ± 0.00	0.00 ± 0.00	0.00 ± 0.00
**4c**	0.0 ± 0.00	0.00 ± 0.00	0.00 ± 0.00
**4d**	0.0 ± 0.00	0.00 ± 0.00	0.00 ± 0.00
Ciprofloxacin hydrochloride	82.73 ± 0.00	3.45 ± 5.97	0.00 ± 0.00

Differences were considered statistically significant when *P* ≤ 0.0007*** by the one‐way analysis of variance and Bonferroni's multiple comparison test.

Considering the toxicity prediction in Table 3, with the exception of compounds **2c–d**, which are potentially hepatotoxic, 4‐alkylamino‐7‐chloroquinolines do not present mutagenic and hepatotoxic potential. On the other hand, 4‐alkylamino‐7‐chloroquinoline–succinimides (**3a–d**) and –phthalimides (**5a–d**) are potentially hepatotoxic. Furthermore, no compound has the potential to cause skin sensitization. Compared to ciprofloxacin, a broad‐spectrum antibiotic used against Gram‐(–) and Gram‐(+) bacteria, it presents mutagenic and hepatotoxic potential. Studies on ciprofloxacin, among other fluoroquinolones, have raised concerns that its use may be associated with an increased risk of aortic aneurysm and dissection [37], among other side effects [38]. Therefore, it is fundamental to seek antibacterial agents that are alternatives to fluoroquinolones.

Given that aquatic organisms are generally the first to be affected by the harmful effects of bioactives and their metabolites, the ecotoxicity of ciprofloxacin hydrochloride was evaluated and classified as moderately toxic to *A. salina* larvae, with an LC_50_ of 185.68 µg·mL^−1^ after 24 h. In another study [39], *A. salina* larvae showed resistance to ciprofloxacin, with an estimated LC_50_> 100 µg·mL^−1^ after 24 h. However, it is important to note the occurrence of certain physical malformations in embryos, although not lethal, within 72 h.

**FIGURE 2:**
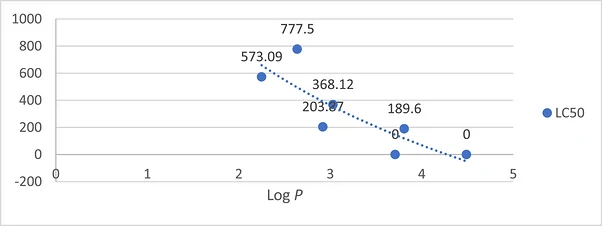
Ecotoxicity–lipophilicity correlation trend for 4‐alkylamino‐7‐chloroquinolines (**2a–g**). Log *P*: predicted octanol/water partition coefficient. LC_50_ (µg·mL^−1^): lethal concentration, where a substance causes the death of 50% of *A. salina* larvae after 24 h.

**FIGURE 3:**
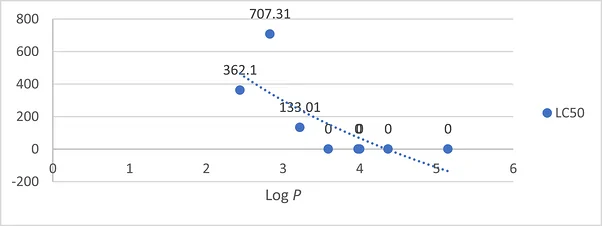
Ecotoxicity–lipophilicity correlation trend for 4‐alkylamino‐7‐chloroquinoline–succinimides (**3a–d**) and –phthalimides (**5a–d**). Log *P*: predicted octanol/water partition coefficient. LC_50_ (µg·mL^−1^): lethal concentration, where a substance causes the death of 50% of *A. salina* larvae after 24 h.

Continuing with the ecotoxicity test, 4‐alkylamino‐7‐chloroquinolines **2a–e** and 4‐alkylamino‐7‐chloroquinoline–succinimides **3a–c** showed slight to moderate toxicity to *A. salina* (LC_50_ 133.01–777.50 µg·mL^−1^), which coincides with the in silico toxicity on *minnow*, whose predicted acute toxicity is not high (log LC_50_ ≥ ‐0.3). A correlation can be observed between ecotoxicity and lipophilicity, which extends to 4‐alkylamino‐7‐chloroquinolines (**2a–g**), 4‐alkylamino‐7‐chloroquinoline–succinimides (**3a–d**) and –phthalimides (**5a–d**), where more lipophilic compounds tend to be more toxic to *A. salina* larvae (Figures 2 and 3).

In a previous study on in silico lipophilicity and in vitro ecotoxicity of alkyl cinnamates [40], the least lipophilic compounds were the least toxic to *A. salina* larvae after 48 h. Thus, it was observed that alkyl groups have a direct influence on the toxic action to *A. salina*. In a more recent in silico study [41], the lipophilicity and ecotoxicity of 2‐chloroacetamides were estimated. The data revealed that the most lipophilic compound, 2‐chloro‐*N*‐cyclohexyl‐*N*‐(1‐phenylpropane‐2‐il)acetamide, exhibited the highest average ecotoxicity for different aquatic species, such as *algae*, *daphnia*, *medaka*, and *minnow*. On the other hand, *N*‐butyl‐2‐chloro‐*N*‐ethylacetamide, which has the least carbon atoms, demonstrated the lowest ecotoxicity. Although studies have been conducted on other nuclei, the observed correlations between lipophilicity and ecotoxicity corroborate the findings of the present work. Furthermore, although *N*‐4‐alkylamino‐7‐chloroquinoline cyclic imides do not enhance the moderate antimicrobial activity of 4‐alkylamino‐7‐chloroquinolines, our results indicate a correlation between increased lipophilicity and higher ecotoxicity.

## 3 Conclusions

In the search for alternative bioactives to AMR and less toxic to the environment, the combination of 4‐alkylamino‐7‐chloroquinolines and cyclic imides was proposed to study the influence of lipophilicity on antibacterial action and ecotoxicity. Thus, seven 4‐alkylamino‐7‐chloroquinolines (**2a–g**) were prepared, of which four were used as precursors (**2a–d**) and reacted with different anhydrides. Using a clean synthetic approach, 4‐alkylamino‐7‐chloroquinoline–succinimides (**3a–d**) and –phthalimides (**5a–d**) were obtained by filtration in distilled water with yields of 60%–85% and 65%–99%, respectively. The best antibacterial results were obtained for 4‐alkylamino‐7‐chloroquinolines (**2b**, **2f**, **2g**), with a MIC of 156.25 µg·mL^−1^ against Gram‐(–) and Gram‐(+) bacteria, while activity was not preserved in *N*‐4‐alkylamino‐7‐chloroquinoline cyclic imides, indicating that 4‐alkylamino‐7‐chloroquinolines linked to succinimide or phthalimide nuclei do not constitute an effective strategy to increase antibacterial activity against the evaluated strains. Combining in silico log *P* and in silico/vitro toxicity data, the results indicate a correlation between increased lipophilicity and higher ecotoxicity, where 4‐alkylamino‐7‐chloroquinolines and 4‐alkylamino‐7‐chloroquinoline–succinimides were, in general, much less toxic than 4‐alkylamino‐7‐chloroquinoline–phthalimides. The toxicity prediction for the most promising 4‐alkylamino‐7‐chloroquinolines (**2b**, **2f**, **2g**) does not represent a potential risk to human health. Furthermore, compound **2b**, which is potentially non‐mutagenic, non‐hepatotoxic, and has no potential to cause skin sensitisation, was the least toxic bioactive to *A. salina* larvae (LC_50_ = 777.50 µg·mL^−1^), thus exhibiting the best toxicological profile.

## 4 Experimental Section

### 4.1 Materials and Methods

All common reagents and solvents were purchased from commercial suppliers and used without further purification. Compounds were synthesized at the Laboratório de Pesquisa em Bioenergia e Síntese Orgânica (LPBS‐UFPB), and melting points were measured in triplicate using a Microquímica MQAPF‐301 instrument (Palhoça, Brazil). ^1^H and ^13^C NMR spectra were acquired at the Laboratório Multiusuário de Caracterização e Análises (LMCA‐UFPB) using Bruker Ascend (Coventry, UK) and Bruker (Billerica, Massachusetts) spectrometers at 400 and 500 MHz for ^1^H, respectively, using 5 mm tubes at 298 K. IR spectra were obtained at the Laboratório de Síntese Orgânica Medicinal (LASOM‐UFPB) using a Shimadzu IRPrestige‐21 spectrometer (Kyoto, Japan) with KBr pellets. HRMS analyses were performed at LMCA‐UFPB using a Shimadzu HPLC (Kyoto, Japan) coupled to a Bruker MicrOTOF II spectrometer (Billerica, Massachusetts) with an electrospray ionization (ESI) source. Acquisition parameters: positive ion polarity, capillary 4500 V, end plate offset –500 V, nebulizer 4.0 bar, dry heater 200°C, dry gas 8.0 L·min^−1^, waste divert valve.

### 4.2 Experimental Procedure for Synthesis of 4‐Alkylamino‐7‐chloroquinolines (**2a–g**)

According to a method previously described by Fiss et al. [7], compounds **2a–g** were obtained in yields ranging from 81% to 100%, whose data were previously described by our research group.

### 4.3 Experimental Procedure for Synthesis of 4‐Alkylamino‐7‐chloroquinoline–Succinimides (**3a–d**) and –Phthalimides (**5a–d**)

A mixture of 4‐alkylamino‐7‐chloroquinoline **2** (3 mmol), succinic or phthalic anhydride (3.1 mmol), and AcOH (3 mL) was maintained under magnetic stirring at 115°C for 24 h (for succinic anhydride) or 6 h (for phthalic anhydride), which was monitored by thin‐layer chromatography (TLC) using methanol in ethyl acetate (MeOH in AcOEt) as eluent. Afterward, the reactional mixture was cooled to room temperature, added to a beaker containing a saturated sodium bicarbonate solution (30 mL), and placed in a freezer until freezing point. Then, the precipitate was filtered and washed with excess distilled water. Compounds (**3a–d** and **5a–d**) were obtained and recrystallized from ethanol in yields ranging from 60% to 99%. Full data for compounds **5a–d** and spectra (Figures S1–36) of compounds (**3a–d** and **5a–d**) are available in the Supporting Information. All compounds are >95% pure by HPLC analysis.

1‐[2‐(7‐Chloroquinolin‐4‐ylamino)ethyl]pyrrolidine‐2,5‐dione (**3a**) in 60% yield; TLC: (10% MeOH in AcOEt, *R*_f_): 0.53 (UV, I_2_); white crystalline solid; mp 253°C–255°C; ^1^H NMR (DMSO‐*d*_6_, 400 MH): δ 8.43 (d, *J* = 5.4 Hz, 1H, H_Ar_), 8.09 (d, *J* = 9.0 Hz, 1H, H_Ar_), 7.80 (d, *J* = 2.2 Hz, 1H, H_Ar_), 7.45 (dd, *J* = 9.0, 2.3 Hz, 1H, H_Ar_), 7.41 (t, *J* = 6.1 Hz, 1H, NH), 6.58 (d, *J* = 5.5 Hz, 1H, H_Ar_), 3.62 (t, *J* = 6.5 Hz, 2H, CH_2_), 3.45 (q, *J* = 6.4 Hz, 2H, CH_2_), 2.60 (s, 4H, 2 × CH_2_) ppm; ^13^C NMR (DMSO‐*d*_6_, 101 MHz): δ 177.8 (C═O), 151.9, 149.8, 149.1, 133.4, 127.5, 124.3, 123.8, 117.5, 98.5 (9 × C_Ar_), 39.4, 36.2, 28.1 (3 × CH_2_) ppm; IR (KBr): *ν* 3201 (N–H), 2924 (H_alkanic_), 1766, 1701 (C═O), 1577, 1489 (C═C), 1334, 1284, 1242, 1203, 1176, 1141, 1080, 1033 (C–N), 817, 767, 702 (Ar), 648 (C–Cl) cm^−1^; HRMS (ESI‐TOF) *m/z*: [M+H]^+^ calcd for C_15_H_15_ClN_3_O_2_: 304.0847; found 304.0848.

1‐[3‐(7‐Chloroquinolin‐4‐ylamino)propyl]pyrrolidine‐2,5‐dione (**3b**) in 85% yield; TLC: (10% MeOH in AcOEt, *R*_f_): 0.42 (UV, I_2_); white crystalline solid; mp 83°C–85°C; ^1^H NMR (DMSO‐*d*_6_, 500 MHz): δ 8.39 (d, *J* = 5.5 Hz, 1H, H_Ar_), 8.23 (d, *J* = 9.0 Hz, 1H, H_Ar_), 7.78 (d, *J* = 2.3 Hz, 1H, H_Ar_), 7.45 (dd, *J* = 9.0, 2.3 Hz, 1H, H_Ar_), 7.26 (t, *J* = 5.6 Hz, 1H, NH), 6.44 (d, *J* = 5.5 Hz, 1H, H_Ar_), 3.49 (t, *J* = 7.0 Hz, 2H, CH_2_), 3.28−3.24 (m, 2H, CH_2_), 2.61 (s, 4H, 2 × CH_2_), 1.88 (p, *J* = 7.0 Hz, 2H, CH_2_) ppm; ^13^C NMR (DMSO‐*d*_6_, 126 MHz): δ 177.8 (C═O), 151.9, 149.9, 149.0, 133.4, 127.5, 124.1, 124.0, 117.4, 98.7 (9 × C_Ar_), 40.0, 36.0, 28.0, 26.0 (4 × CH_2_) ppm; IR (KBr): *ν* 3502, 3356 (N–H), 3082 (H_Ar_), 1762, 1685 (C═O), 1612 (C═N), 1581, 1535, 1489 (C═C), 1350, 1292, 1246, 1184, 1165, 1118, 1083 (C–N), 852, 802, 771, 748 (Ar) cm^−1^; HRMS (ESI‐TOF) *m/z*: [M+H]^+^ calcd for C_16_H_17_ClN_3_O_2_: 318.1004; found 318.1011.

1‐[4‐(7‐Chloroquinolin‐4‐ylamino)butyl]pyrrolidine‐2,5‐dione (**3c**) in 66% yield; TLC: (10% MeOH in AcOEt, *R*_f_): 0.60 (UV, I_2_); white crystalline solid; mp 184°C–186°C; ^1^H NMR (DMSO‐*d*_6_, 400 MHz): δ 8.39 (d, *J* = 5.4 Hz, 1H, H_Ar_), 8.24 (d, *J* = 8.9 Hz, 1H, H_Ar_), 7.78 (d, *J* = 2.2 Hz, 1H, H_Ar_), 7.40 (dd, *J* = 9.0, 2.3 Hz, 1H, H_Ar_), 7.09 (t, *J* = 5.4 Hz, 1H, NH), 6.46 (d, *J* = 5.5 Hz, 1H, H_Ar_), 3.45−3.42 (m, 2H, CH_2_), 3.31−3.26 (m, 2H, CH_2_), 2.61 (s, 4H, 2 × CH_2_), 1.65–1.63 (m, 4H, 2 × CH_2_) ppm; ^13^C NMR (DMSO‐*d*_6_, 101 MHz): *δ* 177.1 (C═O), 151.5, 149.8, 148.8, 133.0, 127.2, 123.6, 123.5, 117.2, 98.3 (9 × C_Ar_), 41.7, 37.3, 27.6, 24.9, 24.6 (5 × CH_2_) ppm; IR (KBr): *ν* 3410 (N–H), 3074 (H_Ar_), 2920, 2858 (H_alkanic_), 1766, 1685 (C═O), 1612 (C═N), 1573, 1535, 1481 (C═C), 1330, 1303, 1246, 1207, 1161, 1138, 1114 (C–N), 829, 810, 763, 732 (Ar), 663 (C–Cl) cm^−1^; HRMS (ESI‐TOF) *m/z*: [M+H]^+^ calcd for C_17_H_19_ClN_3_O_2_: 332.1160; found 332.1173.

1‐[6‐(7‐Chloroquinolin‐4‐ylamino)hexyl]pyrrolidine‐2,5‐dione (**3d**) in 67% yield; TLC: (10% MeOH in AcOEt, *R*_f_): 0.60 (UV, I_2_); pale yellow crystalline solid; mp 84°C–86°C; ^1^H NMR (DMSO‐*d*_6_, 400 MHz): δ 8.37 (d, *J* = 5.4 Hz, 1H, H_Ar_), 8.27 (d, *J* = 9.2 Hz, 1H, H_Ar_), 7.77 (d, *J* = 2.3 Hz, 1H, H_Ar_), 7.42 (dd, *J* = 9.0, 2.3 Hz, 1H, H_Ar_), 7.28 (t, *J* = 5.4 Hz, 1H, NH), 6.43 (d, *J* = 5.5 Hz, 1H, H_Ar_), 3.34 (t, *J* = 7.2 Hz, 2H, CH_2_), 3.23 (q, *J* = 6.8 Hz, 2H, CH_2_), 2.60 (s, 4H, 2 × CH_2_), 1.64−1.61 (m, 2H, CH_2_), 1.48−1.27 (m, 6H, 3 × CH_2_), ppm; ^13^C NMR (DMSO‐*d*_6_, 101 MHz): *δ* 177.7 (C═O), 151.9, 150.0, 149.1, 133.3, 127.4, 124.1, 123.9, 117.4, 98.6 (9 × C_Ar_), 42.3, 37.7, 28.0, 27.6, 27.1, 26.2, 26.0 (7 × CH_2_) ppm; IR (KBr): ν 3525, 3387 (N–H), 2939, 2854 (H_alkanic_), 1766, 1693 (C═O), 1585, 1535 (C═C), 1334, 1284, 1253, 1242, 1192, 1161, 1134, 1080 (C–N), 852, 806, 767, 725 (Ar), 663 (C–Cl) cm^−1^; HRMS (ESI‐TOF) *m/z*: [M+H]^+^ calcd for C_19_H_23_ClN_3_O_2_: 360.1473; found 360.1486.

### 4.4 Antibacterial Assays

In vitro antibacterial assays of 4‐alkylamino‐7‐chloroquinolines (**2a–g**), 4‐alkylamino‐7‐chloroquinoline–succinimides (**3a–d**) and –phthalimides (**5a–d**) were performed at the Laboratório de Biologia Bucal (LABIAL‐UFPB) using strains from the American Type Culture Collection (ATCC): Gram‐(‐) bacteria *K. pneumoniae* ATCC 25922 and *P. aeruginosa* ATCC 27853, and Gram‐(+) bacteria *S. aureus* ATCC 15656 and *S. mutans* ATCC 25175. According to the Clinical and Laboratory Standards Institute (CLSI), document M07‐A10 [42], the assays were conducted using the broth microdilution method to determine the MIC/MBC values. Ciprofloxacin 98% (Sigma‐Aldrich, Brazil) was used as the antibiotic. All experiments were performed in triplicate.

### 4.5 Toxicity Test

The prediction of lipophilicity (log *P*) and toxicity for 4‐alkylamino‐7‐chloroquinolines (**2a–g**), 4‐alkylamino‐7‐chloroquinoline–succinimides (**3a–d**) and –phthalimides (**5a–d**) was calculated using the open‐access electronic site pkCSM “https://biosig.lab.uq.edu.au/pkcsm/prediction (accessed on 8 December 2025)” [35].

In vitro toxicity test of 4‐alkylamino‐7‐chloroquinolines (**2a–g**), 4‐alkylamino‐7‐chloroquinoline–succinimides (**3a–d**) and –phthalimides (**5a–d**) on second‐ and third‐instar (L2–L3) larvae of *A. salina* were performed at LPBS‐UFPB, according to an adapted method reported by Fiss et al. [7]. The cysts of *A. salina* were purchased from Bio Artemia (Grossos, Brazil), and the salt from Instant Ocean Sea Salt (Blacksburg, VA, USA). In a rectangular aquarium of 3 L capacity and using a 20 W light at a height of 20 cm from the water surface, 1 g of *A. salina* cysts was placed in 1 L of saline solution at 30 g·L^−1^, pH 7.5–8.5, and aerated at 25–30°C for 48 h. The toxicity was evaluated at concentrations of 125, 250, and 375 µg·mL^−1^. To this end, stock solutions were prepared at a concentration of 12.5 mg·mL^−1^ for each compound, from which volumes of 50, 100, and 150 µL were captured, added to tubes, and, when necessary, completed with DMSO q.s.p. 150 µL (3%). Next, 100 µL (2%) of Tween 80 was added and completed with saline solution q.s.p. 5 mL. Then, 10 healthy *A. salina* nauplii were introduced into each tube. After 24 h, the number of alive nauplii was counted. Potassium dichromate solutions (125, 250, and 375 µg·mL^−1^) were used as positive control. Two negative controls were prepared, one containing only saline solution and another containing saline solution with 3% DMSO and 2% Tween 80. All experiments were performed in triplicate. The percentage lethality of *A. salina* (%LAS) of the samples was determined by the formula %LAS = [(number of dead nauplii in the test—number of dead nauplii in the negative control)/number of alive nauplii in the negative control] × 100. LC_50_ values were estimated using a linear regression equation in Microsoft Excel for Windows (Table S1). Average percentages of alive *A. salina* nauplii at different concentrations were calculated using GraphPad Prism 5.0 (San Diego, CA, USA) (Table 4). The statistic was analyzed by ANOVA method at a 95% confidence level (Figure S37).

## Author Contributions

Conceptualization: G.F.F.; Data curation: A.P.L.‐F., T.C.S., M.B.S., and H.D.S.S.; Formal analysis: G.F.F.; Funding acquisition: G.F.F. and P.F.A.‐F.; Investigation: G.F.F.; Methodology: A.A;‐J. and F.C.S.; Project administration: G.F.F.; Resources: G.F.F. and P.F.A.‐F.; Supervision: G.F.F. and P.F.A.‐F.; Validation: A.P.S.; Visualization: G.F.F.; Writing – original draft: G.F.F.; Writing – review and editing: G.F.F., H.D.S.S., and P.S.V.L.

## Conflicts of Interest

The authors declare no conflicts of interest.

## Supporting Information

Supporting Information for this article is available on the WWW under https://doi.org/10.1002/cbdv.71688.

## Supporting information




**Supporting File 1**: cbdv71688‐sup‐0001‐SuppMat.pdf.

## Data Availability

The data that supports the findings of this study are available in the supplementary material of this article
