# Supplementary material for: Clean Synthesis of N‐4‐Alkylamino‐7‐chloroquinoline Cyclic Imides: Influence of Lipophilicity on Antibacterial and Ecotoxicological Properties
Source: Chem Biodivers. 2026 Sep 10;23(9):e71688. doi: 10.1002/cbdv.71688 (PMC13559871; doi:10.1002/cbdv.71688)
Supplement: Supplementary file 1 [file CBDV-23-e71688-s001.pdf]

# Clean Synthesis of *N*-4-Alkylamino-7-chloroquinoline Cyclic Imides: Influence of Lipophilicity on Antibacterial and Ecotoxicological Properties

Agenor P. Luz-Filho,[a] Thais C. Silva, [a] Matheus B. Silva,[a] Abraão P. Sousa,[a] Helivaldo D. S. Souza,[a] Priscila S. V. Lima,[b] Alexandre Almeida-Júnior,[c] Fábio C. Sampaio,[c] Petrônio F. Athayde-Filho,[a] and Gabriela F. Fiss*,[a]

[a] Laboratório de Pesquisa em Bioenergia e Síntese Orgânica (LPBS); Department of Chemistry, Universidade Federal da Paraíba (UFPB), João Pessoa 58051-900, Brazil, gffiss@gmail.com, gff@academico.ufpb.br

[b] Núcleo de Química de Heterociclos (NUQUIMHE), Department of Chemistry, Universidade Federal de Santa Maria, Santa Maria 97105-900, Brazil

[c] Laboratório de Biologia Bucal (LABIAL), Department of Clinical and Community Dentistry, UFPB, João Pessoa 58051-900, Brazil

## Supporting Information

Full Data for 4-Alkylamino-7-chloroquinoline–phthalimides (**5a–d**)

2-[2-(7-Chloroquinolin-4-ylamino)ethyl]isoindoline-1,3-dione (**5a**) in 65% yield; white crystalline solid; mp 243–244 °C (lit. [27] mp 200–203 °C); $^1$H NMR (400 MHz, DMSO-$d_6$): δ 8.42 (d, *J* = 5.4 Hz, 1H, $H_{Ar}$), 8.05 (d, *J* = 9.1 Hz, 1H, $H_{Ar}$), 7.86–7.81 (m, 4H, $H_{Ar}$), 7.78 (d, *J* = 2.2 Hz, 1H, $H_{Ar}$), 7.49 (t, *J* = 6.1 Hz, 1H, NH), 7.41 (dd, *J* = 9.0, 2.2 Hz, 1H, $H_{Ar}$), 6.63 (d, *J* = 5.5 Hz, 1H, $H_{Ar}$), 3.83 (t, *J* = 6.2 Hz, 2H, $CH_2$), 3.59 (q, *J* = 6.1 Hz, 2H, $CH_2$) ppm; $^{13}$C NMR (101 MHz, DMSO-$d_6$): δ 167.9 (C=O), 151.8, 149.8, 149.1, 134.3, 133.4, 131.7, 127.5, 124.2, 123.8, 122.9, 117.5, 98.5 (12 × $C_{Ar}$), 40.1, 35.8 (2 × $CH_2$) ppm; HRMS (ESI-TOF) *m/z*: $[M+H]^+$ calcd for $C_{19}H_{16}ClN_3O_2$: 352.0847; found 352.0834.

2-[3-(7-Chloroquinolin-4-ylamino)propyl]isoindoline-1,3-dione (**5b**) in 99% yield; white crystalline solid; mp 177–178 °C (lit. [27] mp 165–167 °C); $^1$H NMR (400 MHz, DMSO-$d_6$): δ 8.37 (d, *J* = 5.4 Hz, 1H, $H_{Ar}$), 8.19 (d, *J* = 9.1 Hz, 1H, $H_{Ar}$), 7.84–7.79 (m, 4H, $H_{Ar}$), 7.76 (d, *J* = 2.2 Hz, 1H, $H_{Ar}$), 7.41 (dd, *J* = 9.0, 2.3 Hz, 1H, $H_{Ar}$), 7.26 (t, *J* = 5.3 Hz, 1H, NH), 6.45 (d, *J* = 5.5 Hz, 1H, $H_{Ar}$), 3.72 (t, *J* = 6.9 Hz, 2H, $CH_2$), 3.35–3.30 (m, 2H, $CH_2$), 2.02 (p, *J* = 7.0 Hz, 2H, $CH_2$) ppm; $^{13}$C NMR (101 MHz, DMSO-$d_6$): δ 167.9 (C=O), 151.8, 149.9, 149.0, 134.2, 133.3, 131.6, 127.4, 124.0, 123.9, 122.9, 117.4, 98.7 (12 × $C_{Ar}$), 40.1, 35.6, 26.7 (3 × $CH_2$) ppm; HRMS (ESI-TOF) *m/z*: $[M+H]^+$ calcd for $C_{20}H_{18}ClN_3O_2$: 366.1004; found 366.1000.

2-[4-(7-Chloroquinolin-4-ylamino)butyl]isoindoline-1,3-dione (**5c**) in 76% yield; white crystalline solid; mp 156–157 °C (lit. [17, 18] mp 115–116 °C); $^1$H NMR (500 MHz, DMSO-$d_6$): δ 8.35 (d, *J* = 5.4 Hz, 1H, $H_{Ar}$), 8.23 (d, *J* = 9.0 Hz, 1H, $H_{Ar}$), 7.84–7.80 (m, 4H, $H_{Ar}$), 7.75 (d, *J* = 2.1 Hz, 1H, $H_{Ar}$), 7.41 (dd, *J* = 9.0, 2.2 Hz, 1H, $H_{Ar}$), 7.29 (t, *J* = 5.1 Hz, 1H, NH), 6.45 (d, *J* = 5.5 Hz, 1H, $H_{Ar}$), 3.63 (t, *J* = 6.6 Hz, 2H, $CH_2$), 3.28 (q, *J* = 6.4 Hz, 2H, $CH_2$), 1.76–1.64 (m, 4H, 2 × $CH_2$) ppm; $^{13}$C NMR (126 MHz, DMSO-$d_6$): δ 167.9 (C=O), 151.6, 150.1, 148.8, 134.3, 133.4, 131.5, 127.2, 124.0, 123.9, 122.9, 117.3, 98.6 (12 × $C_{Ar}$), 41.9, 37.1, 25.6, 25.1 (4 × $CH_2$) ppm; HRMS (ESI-TOF) *m/z*: $[M+H]^+$ calcd for $C_{21}H_{19}ClN_3O_2$: 380.1160; found 380.1154.

2-[6-(7-Chloroquinolin-4-ylamino)hexyl]isoindoline-1,3-dione (**5d**) in 90% yield; pale yellow crystalline solid; mp 142–144 °C (lit. [17, 18] mp 109–110 °C); $^1$H NMR (400 MHz, DMSO-$d_6$): δ 9.02 (t, *J* = 4.9 Hz, 1H, NH), 8.56 (d, *J* = 9.1 Hz, 1H, $H_{Ar}$), 8.44 (d, *J* = 6.7 Hz, 1H, $H_{Ar}$), 7.98 (d, *J* = 2.1 Hz, 1H, $H_{Ar}$), 7.83–7.80 (m, 4H, $H_{Ar}$), 7.62 (dd, *J* = 9.1, 2.2 Hz, 1H, $H_{Ar}$), 6.71 (d, *J* = 6.8 Hz, 1H, $H_{Ar}$), 3.55 (t, *J* = 7.1 Hz, 2H, $CH_2$), 3.41 (q, *J* = 6.8 Hz, 2H, $CH_2$), 1.67–1.57 (m, 4H, 2 × $CH_2$), 1.41–1.29 (m, 4H, 2 × $CH_2$) ppm; $^{13}$C NMR (101 MHz, DMSO-$d_6$): δ 167.9 (C=O), 153.9, 144.8, 141.0, 136.7, 134.3, 131.5, 125.9, 125.4, 122.9, 121.0, 115.9, 98.4 (12 × $C_{Ar}$), 42.8, 37.3, 27.8, 27.4, 26.0, 25.9 (6 × $CH_2$) ppm; HRMS (ESI-TOF) *m/z*: $[M+H]^+$ calcd for $C_{23}H_{24}ClN_3O_2$: 480.1473; found 408.1460.

## Full Spectra for 4-Alkylamino-7-chloroquinoline–succinamides (**3a–d**)

**Figure S1.** $^{1}H$ NMR spectrum (DMSO-$d_6$, 400 MHz) of compound **3a**.
**Figure S2.** $^{13}C$ NMR spectrum (DMSO-$d_6$, 101 MHz) of compound **3a**.
**Figure S3.** IR spectrum (KBr) of compound **3a**.
**Figure S4.** HRMS spectrum (ESI) of compound **3a**.
**Figure S5.** HPLC chromatogram of compound **3a**.
**Figure S6.** $^{1}H$ NMR spectrum (DMSO-$d_6$, 500 MHz) of compound **3b**.
**Figure S7.** $^{13}C$ NMR spectrum (DMSO-$d_6$, 126 MHz) of compound **3b**.
**Figure S8.** IR spectrum (KBr) of compound **3b**.
**Figure S9.** HRMS spectrum (ESI) of compound **3b**.
**Figure S10.** HPLC chromatogram of compound **3b**.
**Figure S11.** $^{1}H$ NMR spectrum (DMSO-$d_6$, 400 MHz) of compound **3c**.
**Figure S12.** $^{13}C$ NMR spectrum (DMSO-$d_6$, 101 MHz) of compound **3c**.
**Figure S13.** IR spectrum (KBr) of compound **3c**.
**Figure S14.** HRMS spectrum (ESI) of compound **3c**.
**Figure S15.** HPLC chromatogram of compound **3c**.
**Figure S16.** $^{1}H$ NMR spectrum (DMSO-$d_6$, 400 MHz) of compound **3d**.
**Figure S17.** $^{13}C$ NMR spectrum (DMSO-$d_6$, 101 MHz) of compound **3d**.
**Figure S18.** IR spectrum (KBr) of compound **3d**.
**Figure S19.** HRMS spectrum (ESI) of compound **3d**.
**Figure S20.** HPLC chromatogram of compound **3d**.

## Full Spectra for 4-Alkylamino-7-chloroquinoline–phthalimides (**5a–d**)

**Figure S21.** $^{1}H$ NMR spectrum (DMSO-$d_6$, 400 MHz) of compound **5a**.
**Figure S22.** $^{13}C$ NMR spectrum (DMSO-$d_6$, 101 MHz) of compound **5a**.
**Figure S23.** HRMS spectrum (ESI) of compound **5a**.
**Figure S24.** HPLC chromatogram of compound **5a**.
**Figure S25.** $^{1}H$ NMR spectrum (DMSO-$d_6$, 400 MHz) of compound **5b**.
**Figure S26.** $^{13}C$ NMR spectrum (DMSO-$d_6$, 101 MHz) of compound **5b**.
**Figure S27.** HRMS spectrum (ESI) of compound **5b**.
**Figure S28.** HPLC chromatogram of compound **5b**.
**Figure S29.** $^{1}H$ NMR spectrum (DMSO-$d_6$, 500 MHz) of compound **5c**.
**Figure S30.** $^{13}C$ NMR spectrum (DMSO-$d_6$, 126 MHz) of compound **5c**.
**Figure S31.** HRMS spectrum (ESI) of compound **5c**.
**Figure S32.** HPLC chromatogram of compound **5c**.
**Figure S33.** $^{1}H$ NMR spectrum (DMSO-$d_6$, 400 MHz) of compound **5d**.
**Figure S34.** $^{13}C$ NMR spectrum (DMSO-$d_6$, 101 MHz) of compound **5d**.
**Figure S35.** HRMS spectrum (ESI) of compound **5d**.
**Figure S36.** HPLC chromatogram of compound **5d**.

## Ecotoxicity Data for 4-Alkylamino-7–chloroquinoline **2d**, 4-Alkylamino-7-chloroquinoline–succinamides **3a–c** and Ciprofloxacin Hydrochloride

**Table S1.** Estimated 50% lethal concentration ($LC_{50}$) of compounds **2d**, **3a–c**, and ciprofloxacin hydrochloride on *Artemia salina* larvae after 24 h.
**Figure S37.** Average percentages of alive *A. salina* nauplii in different concentrations of compounds **2d**, **3a–c**, and ciprofloxacin hydrochloride after 24 h. Differences were considered statistically significant when P ≤ 0.0007*** using one-way analysis of variance and Bonferroni's Multiple Comparison Test.

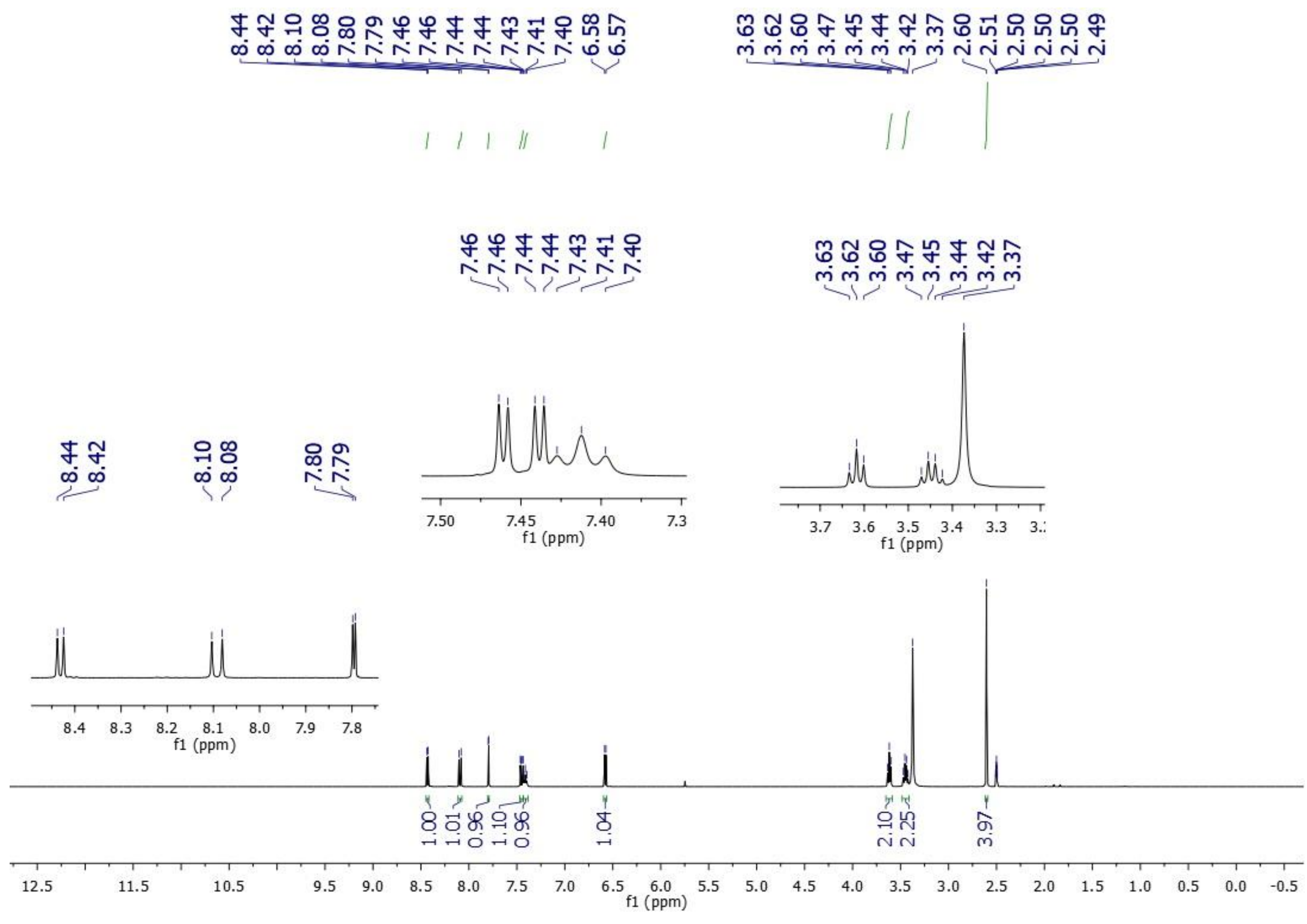

**Figure S1.** $^{1}H$ NMR spectrum (DMSO-$d_6$, 400 MHz) of compound **3a**.

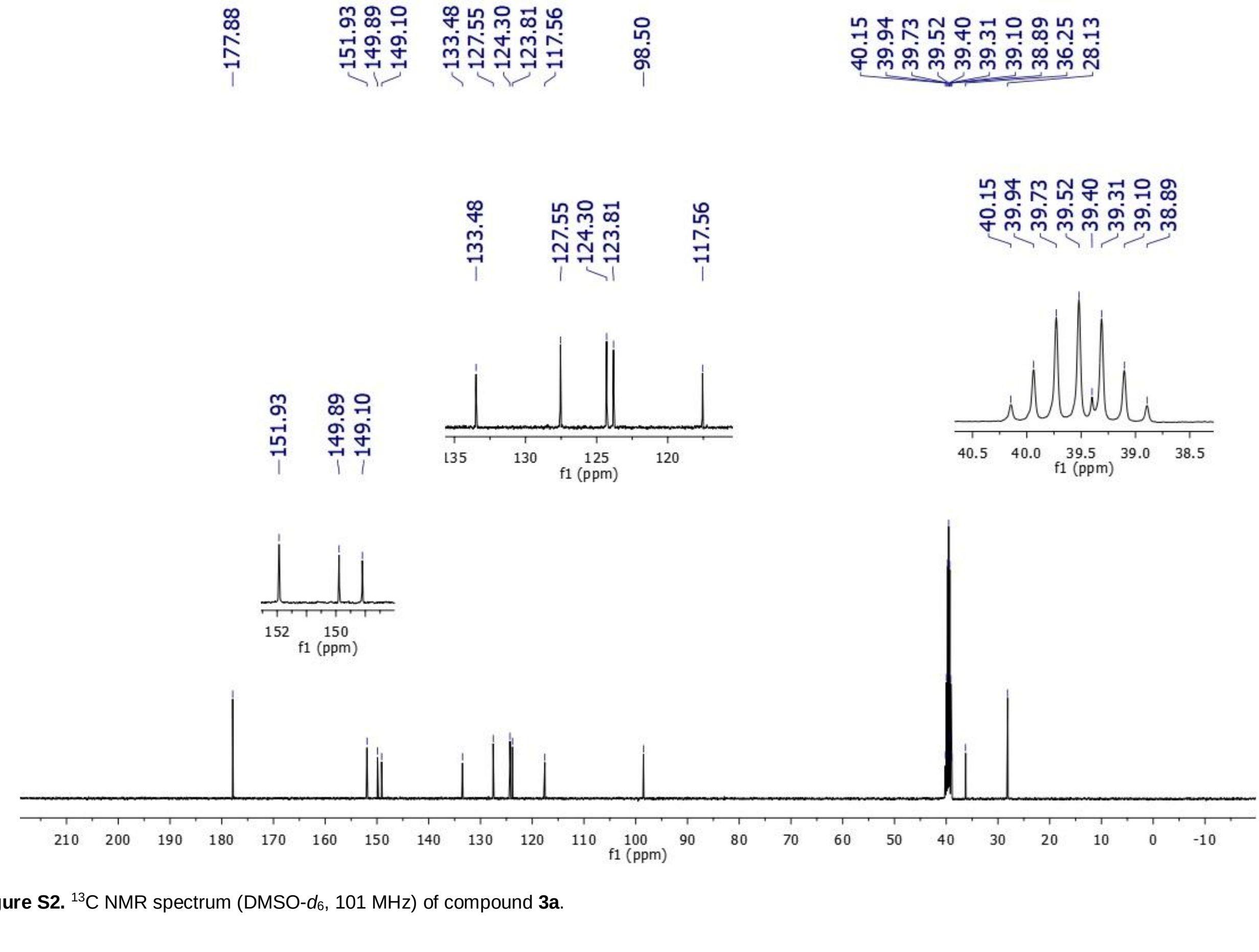

**Figure S2.** $^{13}C$ NMR spectrum (DMSO-$d_6$, 101 MHz) of compound **3a**.

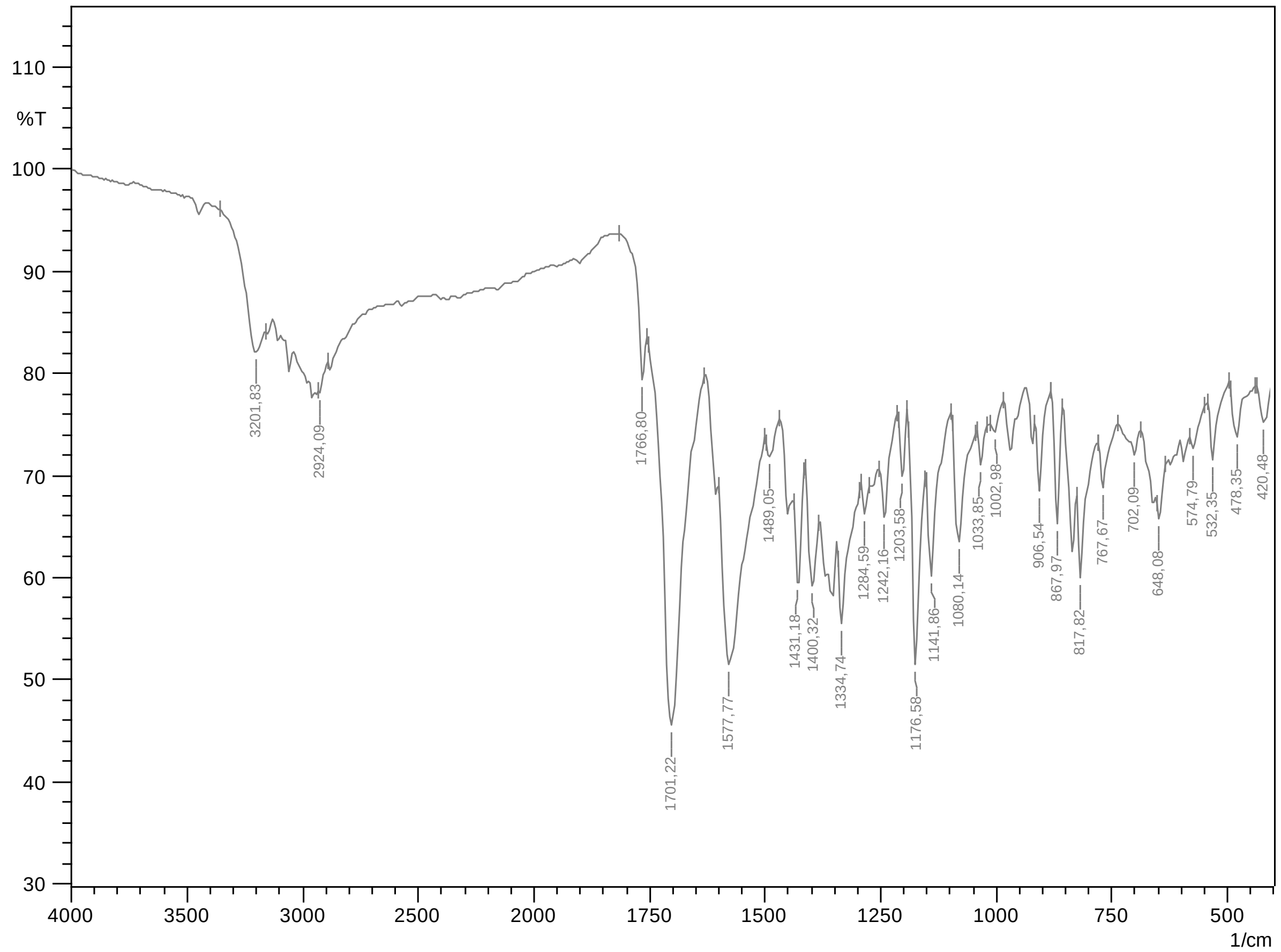


**Figure S3.** IR spectrum (KBr) of compound **3a**.

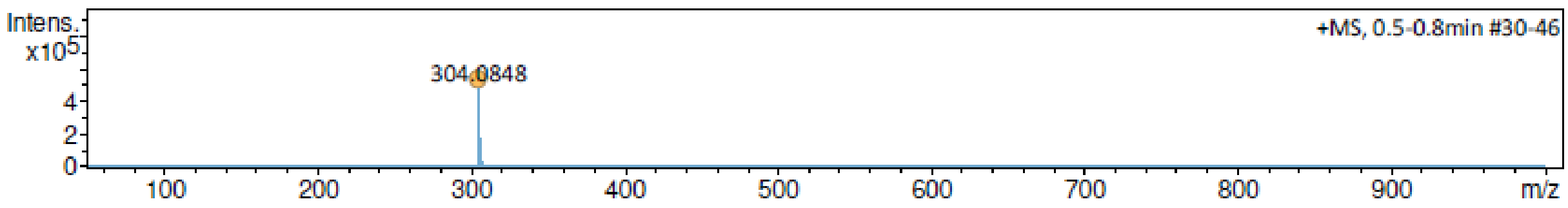


| Meas. m/z | # | Ion Formula | m/z | err [ppm] | mSigma | # mSigma | Score | rdb | e⁻ Conf | N-Rule |
|---|---|---|---|---|---|---|---|---|---|---|
| 304.0848 | 1 | C15H15ClN3O2 | 304.0847 | -0.2 | 3.9 | 1 | 100.00 | 9.5 | even | ok |

**Figure S4.** HRMS spectrum (ESI) of compound **3a**.

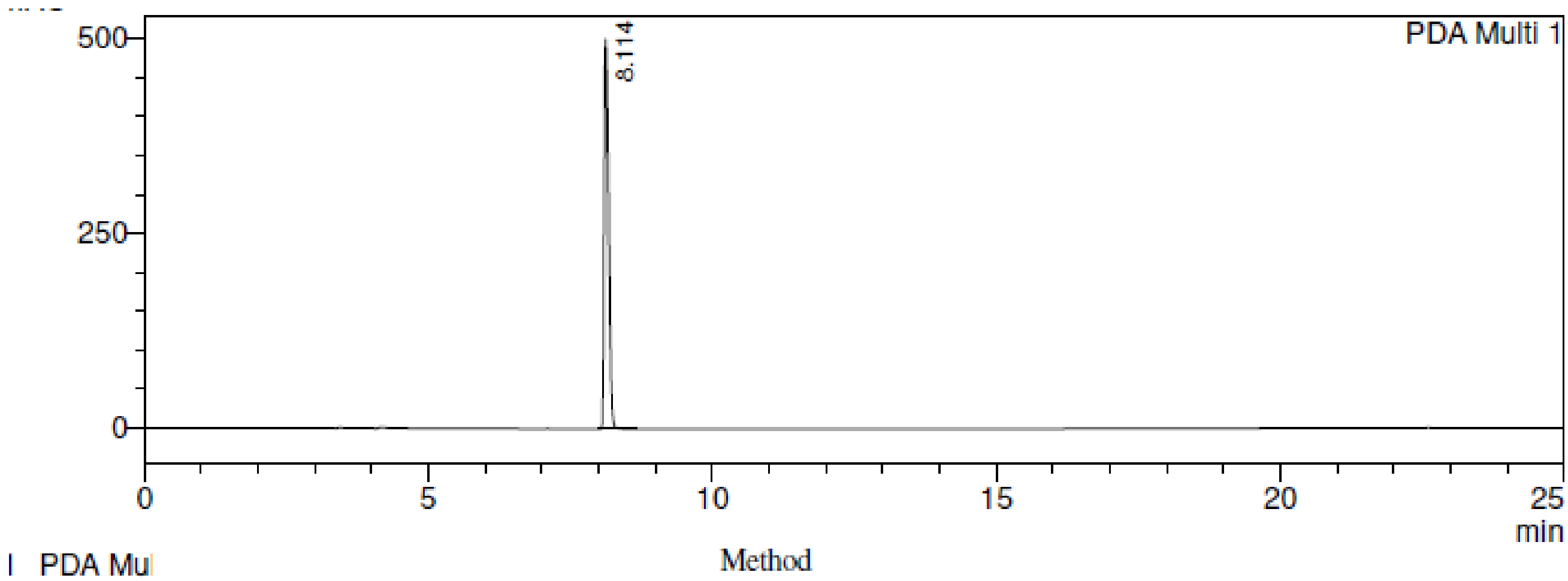


Method

<<Comment>>

<<LC Program>>

| Time | Unit | Command | Value |
|---|---|---|---|
| 0.01 | Pumps | B.Conc | 5 |
| 20.00 | Pumps | B.Conc | 100 |
| 25.00 | Pumps | B.Conc | 100 |
| 26.00 | Pumps | B.Conc | 5 |
| 46.00 | Controller | Stop | |

PeakTable

PDA Ch1 343nm 4nm

| Peak# | Ret. Time | Area | Area % |
|---|---|---|---|
| 1 | 8.114 | 2654498 | 100.000 |
| Total | | 2654498 | 100.000 |

Purity

Peak# : 1
Retention Time : 8.114
Compound Name :

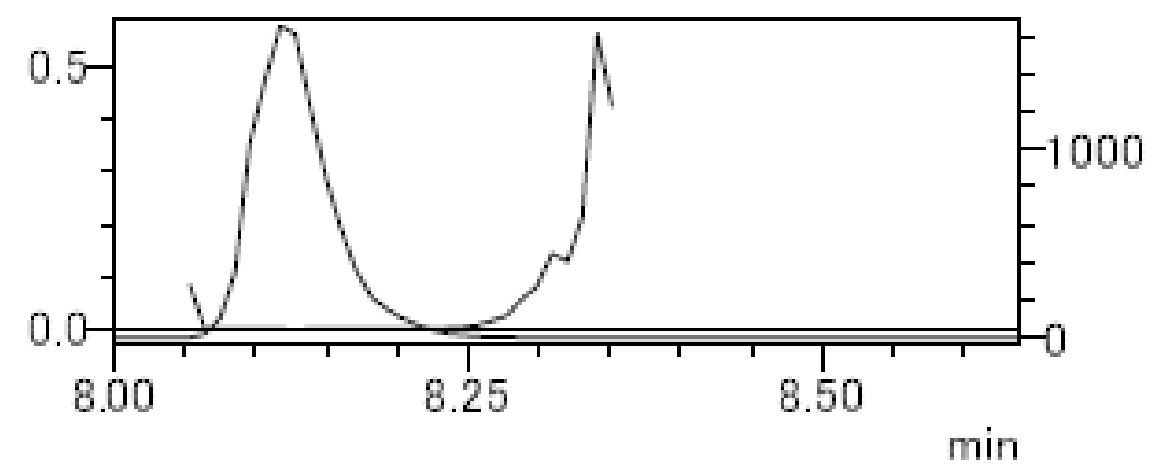


Impurity :Detected at 8.13 min
Peak purity index : 0.999100
Single point threshold : 0.999969
Minimum peak purity index : -868

**Figure S5.** HPLC chromatogram of compound **3a**.

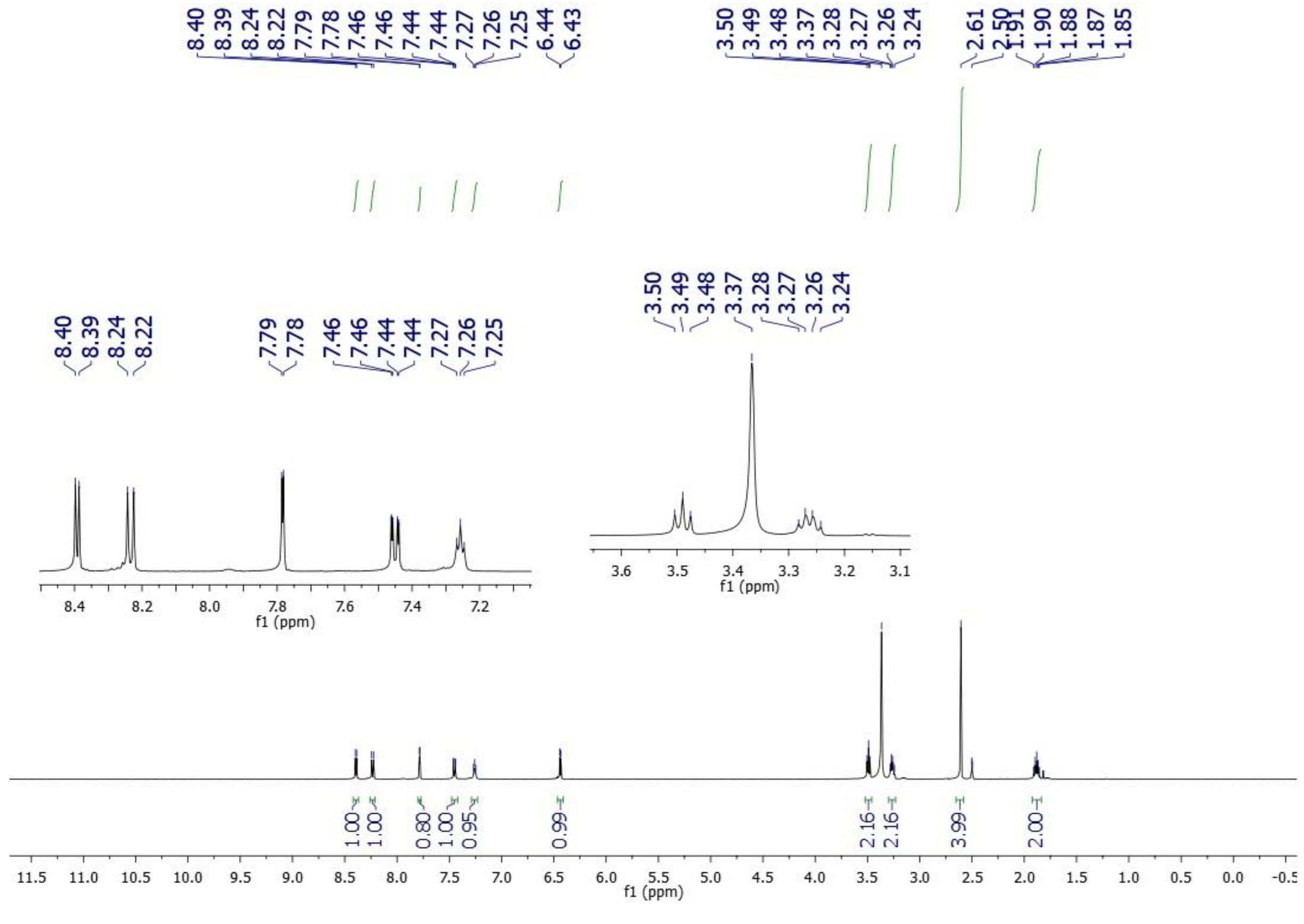

**Figure S6.** $^{1}H$ NMR spectrum (DMSO-$d_6$, 500 MHz) of compound **3b**.

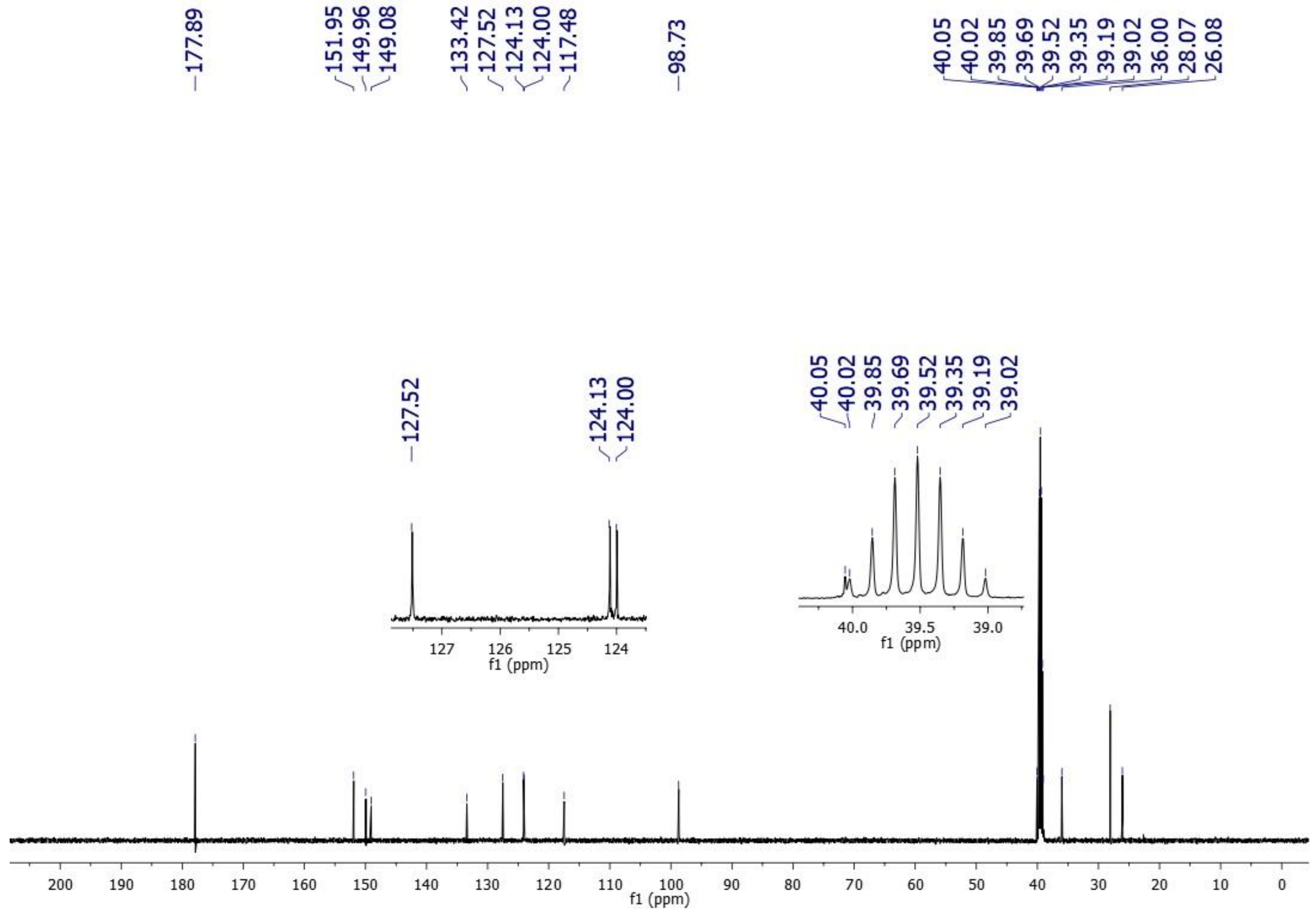

**Figure S7.** $^{13}C$ NMR spectrum (DMSO-$d_6$, 126 MHz) of compound **3b**.

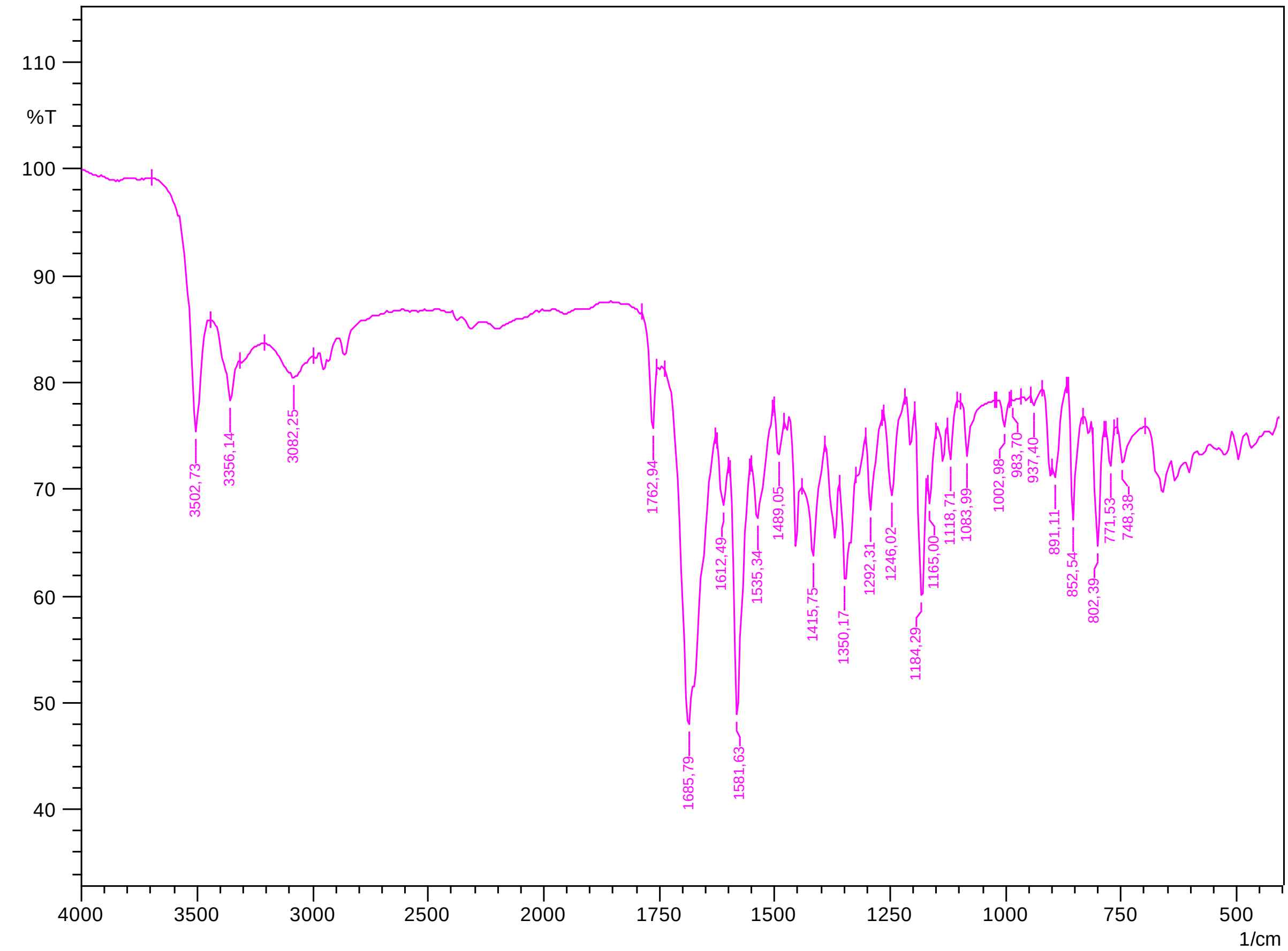


**Figure S8.** IR spectrum (KBr) of compound **3b**.

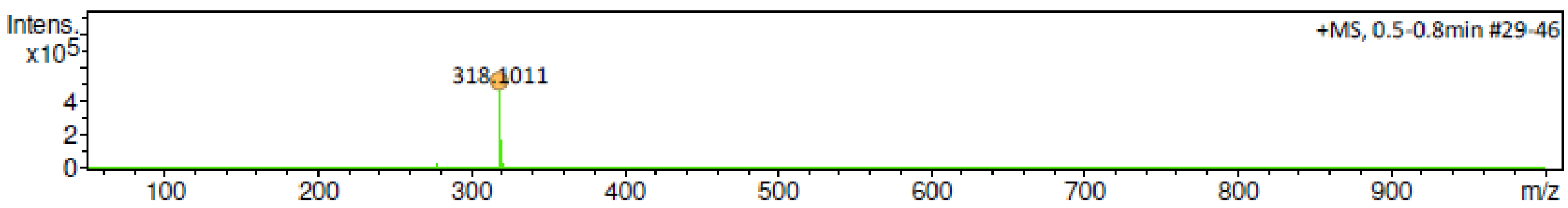


| Meas. m/z | # | Ion Formula | m/z | err [ppm] | mSigma | # mSigma | Score | rdb | e⁻ Conf | N-Rule |
|---|---|---|---|---|---|---|---|---|---|---|
| 318.1011 | 1 | C16H17ClN3O2 | 318.1004 | -2.3 | 2.2 | 1 | 100.00 | 9.5 | even | ok |

**Figure S9.** HRMS spectrum (ESI) of compound **3b**.

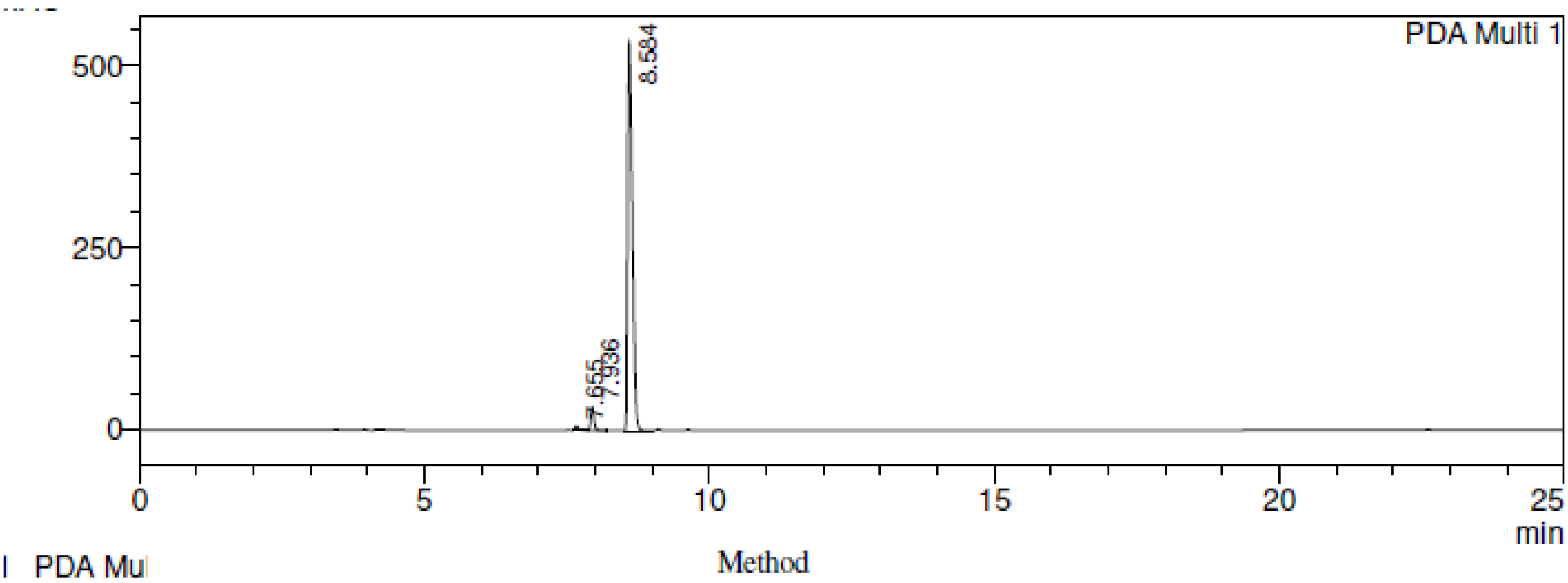


PDA Mul

Method

<<Comment>>

<<LC Program>>

| Time | Unit | Command | Value |
|---|---|---|---|
| 0.01 | Pumps | B.Conc | 5 |
| 20.00 | Pumps | B.Conc | 100 |
| 25.00 | Pumps | B.Conc | 100 |
| 26.00 | Pumps | B.Conc | 5 |
| 46.00 | Controller | Stop | |

PeakTable

PDA Ch1 343nm 4nm

| Peak# | Ret. Time | Area | Area % |
|---|---|---|---|
| 1 | 7.655 | 25593 | 0.779 |
| 2 | 7.936 | 132248 | 4.028 |
| 3 | 8.584 | 3125623 | 95.193 |
| Total | | 3283464 | 100.000 |

Purity

Peak# : 3
Retention Time : 8.584
Compound Name :

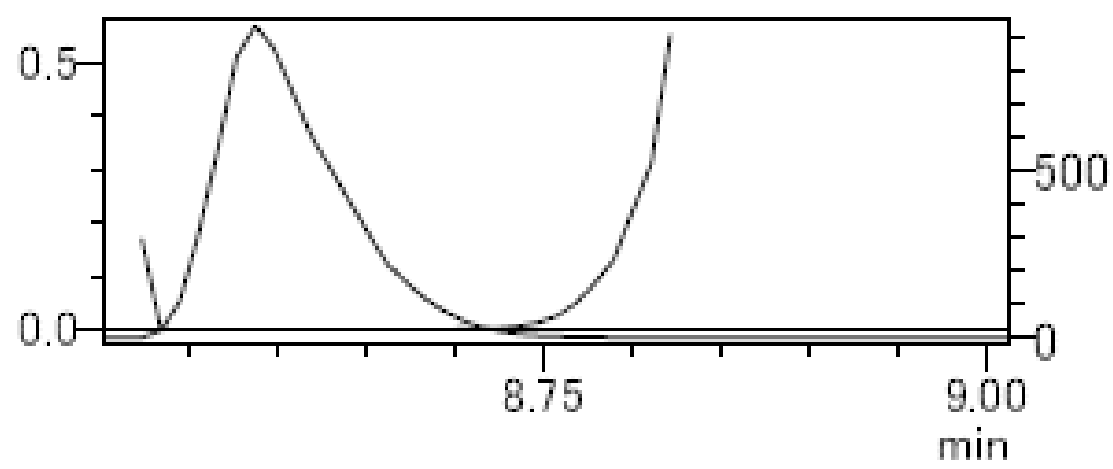


Impurity :Detected at 8.60 min
Peak purity index : 0.999954
Single point threshold : 0.999965
Minimum peak purity index : -10

**Figure S10.** HPLC chromatogram of compound **3b**.

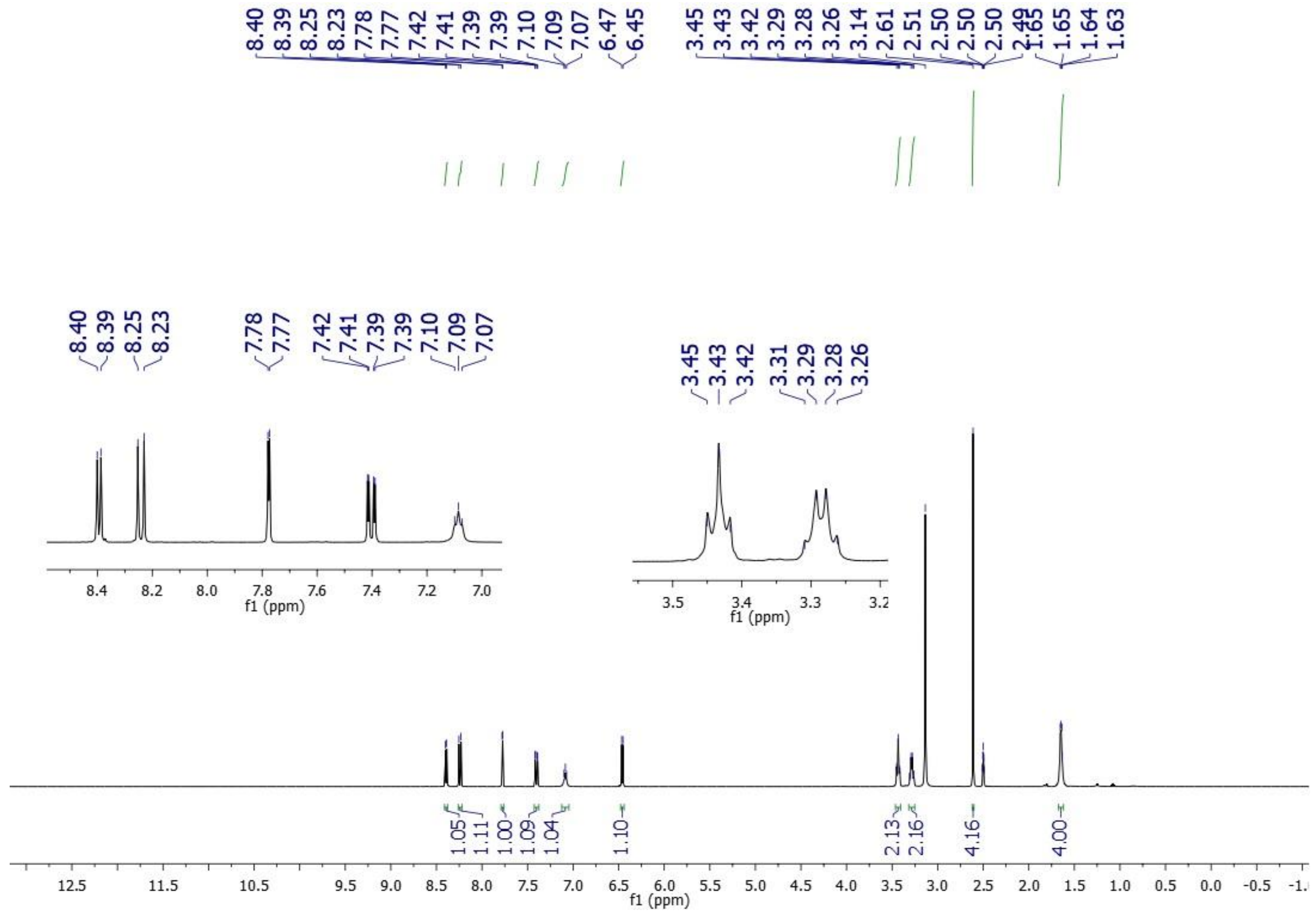

**Figure S11.** $^{1}H$ NMR spectrum (DMSO-$d_6$, 400 MHz) of compound **3c**.

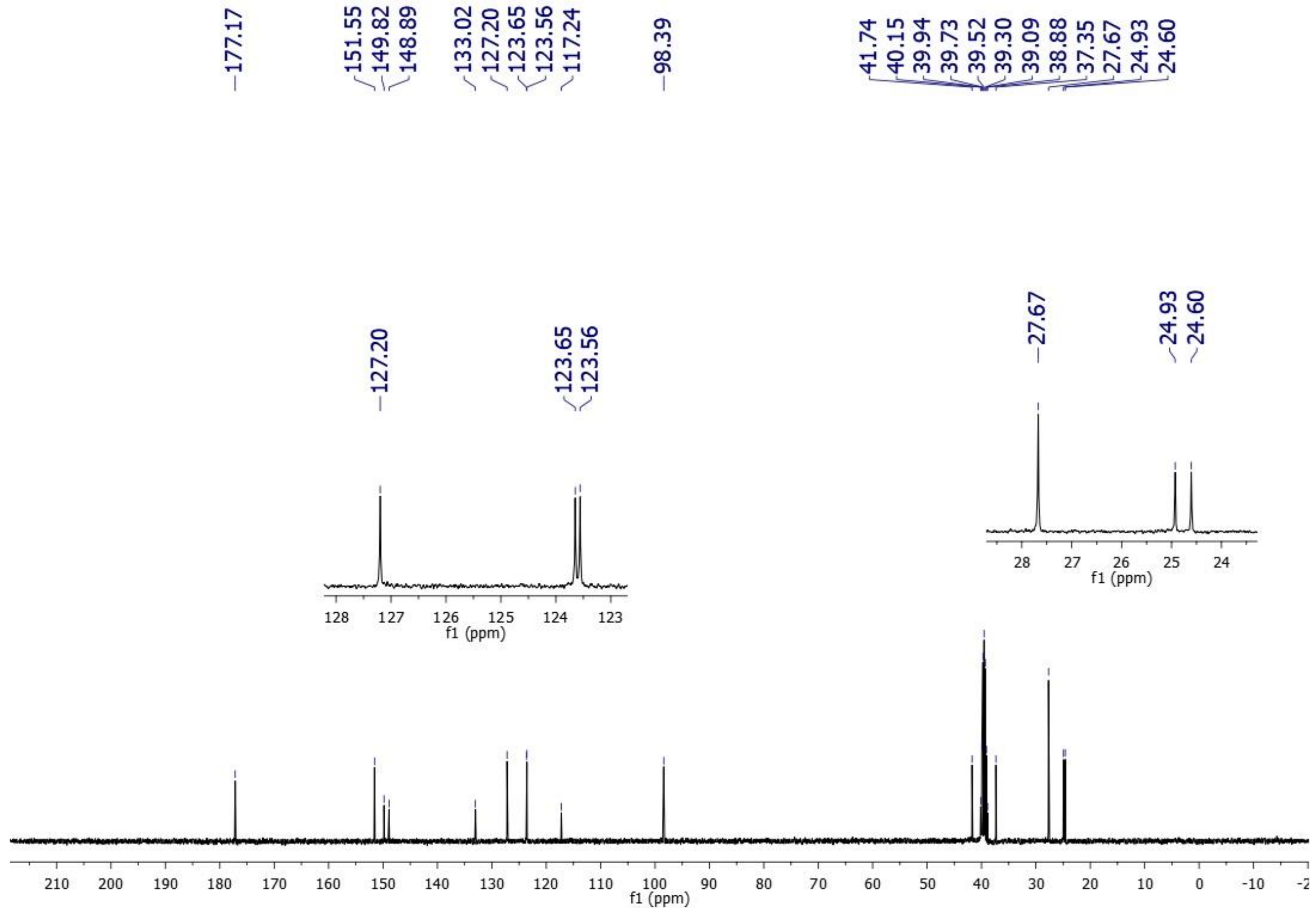

**Figure S12.** $^{13}C$ NMR spectrum (DMSO-$d_6$, 101 MHz) of compound **3c**.

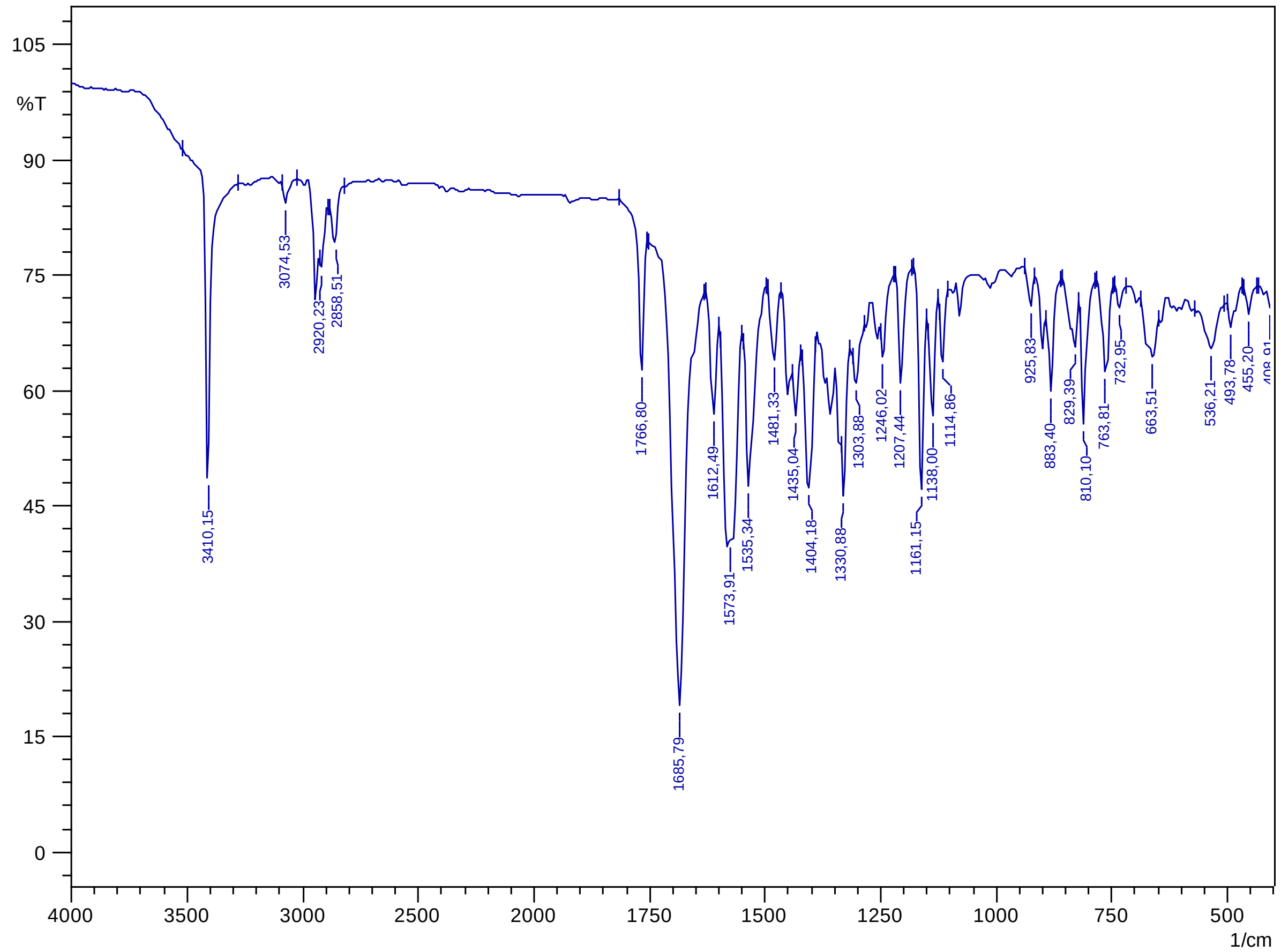


**Figure S13.** IR spectrum (KBr) of compound **3c**.

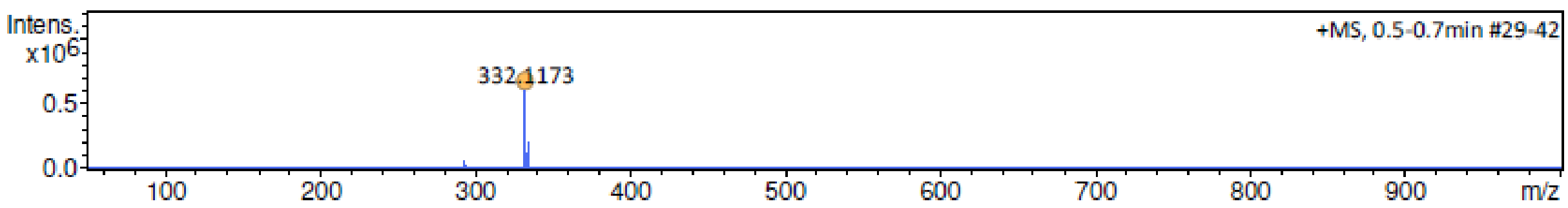


| Meas. m/z | # | Ion Formula | m/z | err [ppm] | mSigma | # mSigma | Score | rdb | e⁻ Conf | N-Rule |
|---|---|---|---|---|---|---|---|---|---|---|
| 332.1173 | 1 | C17H19ClN3O2 | 332.1160 | -3.7 | 4.4 | 1 | 100.00 | 9.5 | even | ok |

**Figure S14.** HRMS spectrum (ESI) of compound **3c**.

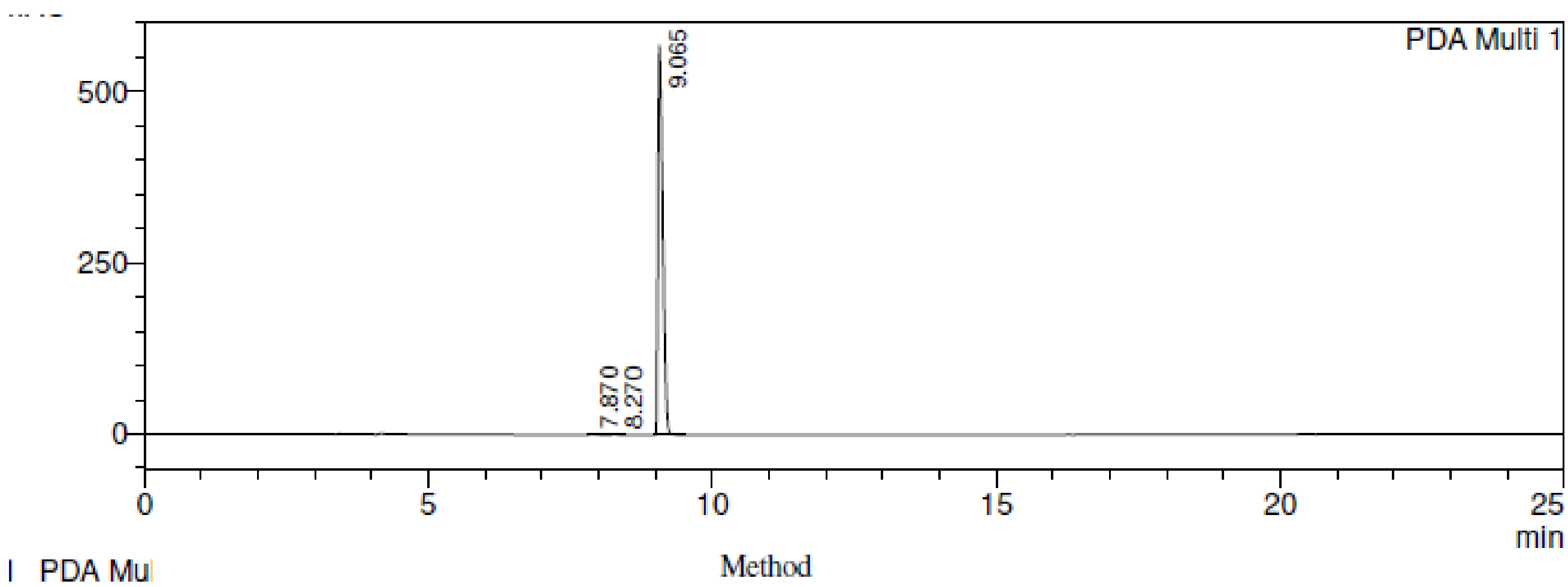


Method

<<Comment>>

<<LC Program>>

| Time | Unit | Command | Value |
|---|---|---|---|
| 0.01 | Pumps | B.Conc | 5 |
| 20.00 | Pumps | B.Conc | 100 |
| 25.00 | Pumps | B.Conc | 100 |
| 26.00 | Pumps | B.Conc | 5 |
| 46.00 | Controller | Stop | |

PeakTable

PDA Ch1 343nm 4nm

| Peak# | Ret. Time | Area | Area % |
|---|---|---|---|
| 1 | 7.870 | 5230 | 0.153 |
| 2 | 8.270 | 5055 | 0.148 |
| 3 | 9.065 | 3406230 | 99.699 |
| Total | | 3416514 | 100.000 |

Purity

Peak# : 3
Retention Time : 9.065
Compound Name :

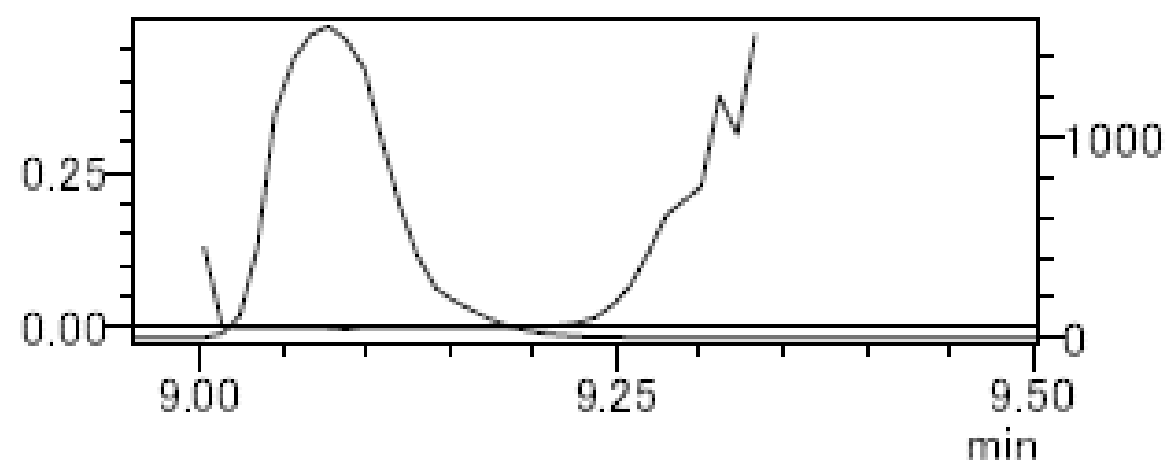


Impurity :Detected at 9.09 min
Peak purity index : 0.996844
Single point threshold : 0.999969
Minimum peak purity index : -3125

**Figure S15.** HPLC chromatogram of compound **3c**.

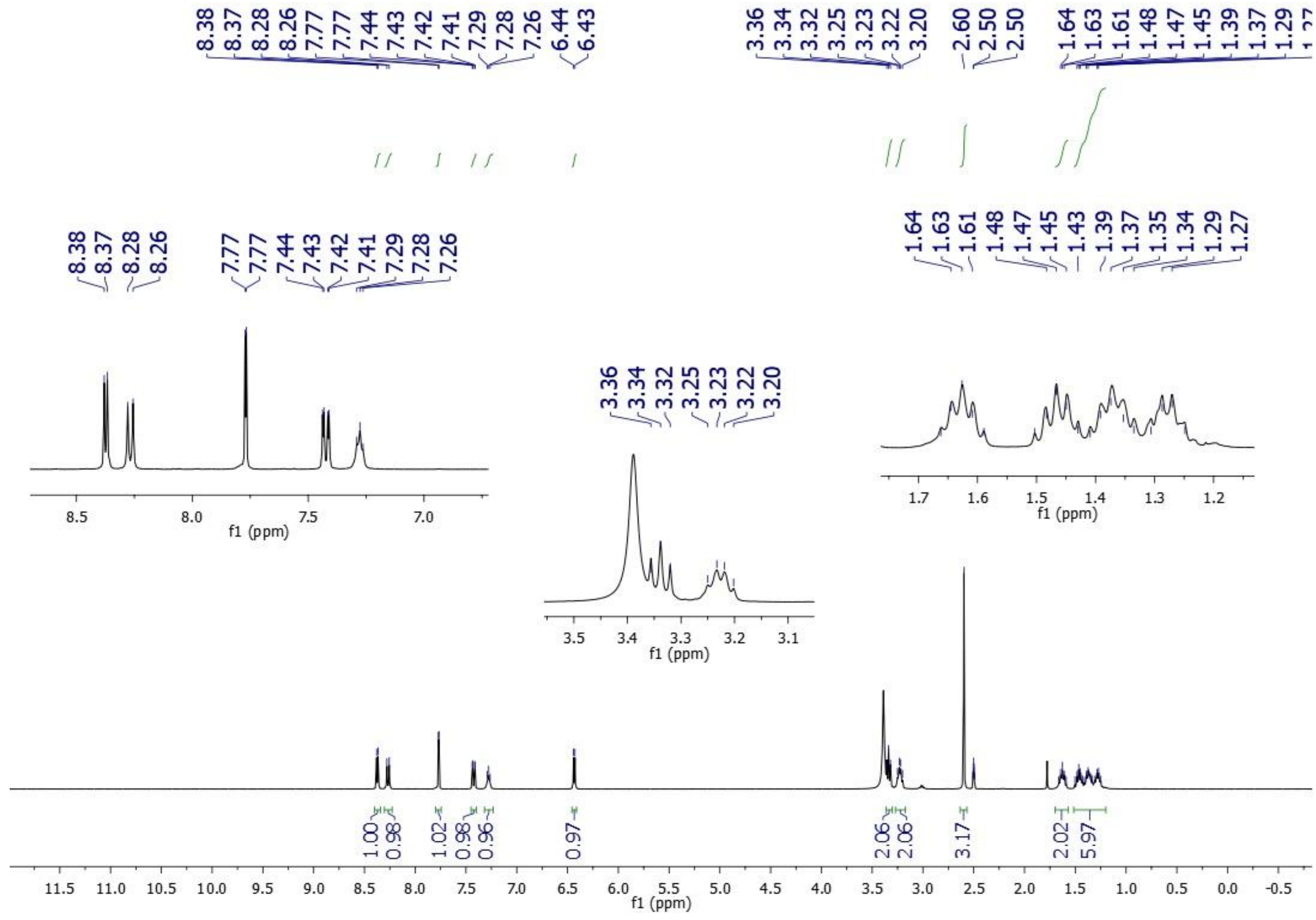

**Figure S16.** $^{1}H$ NMR spectrum (DMSO-$d_6$, 400 MHz) of compound **3d**.

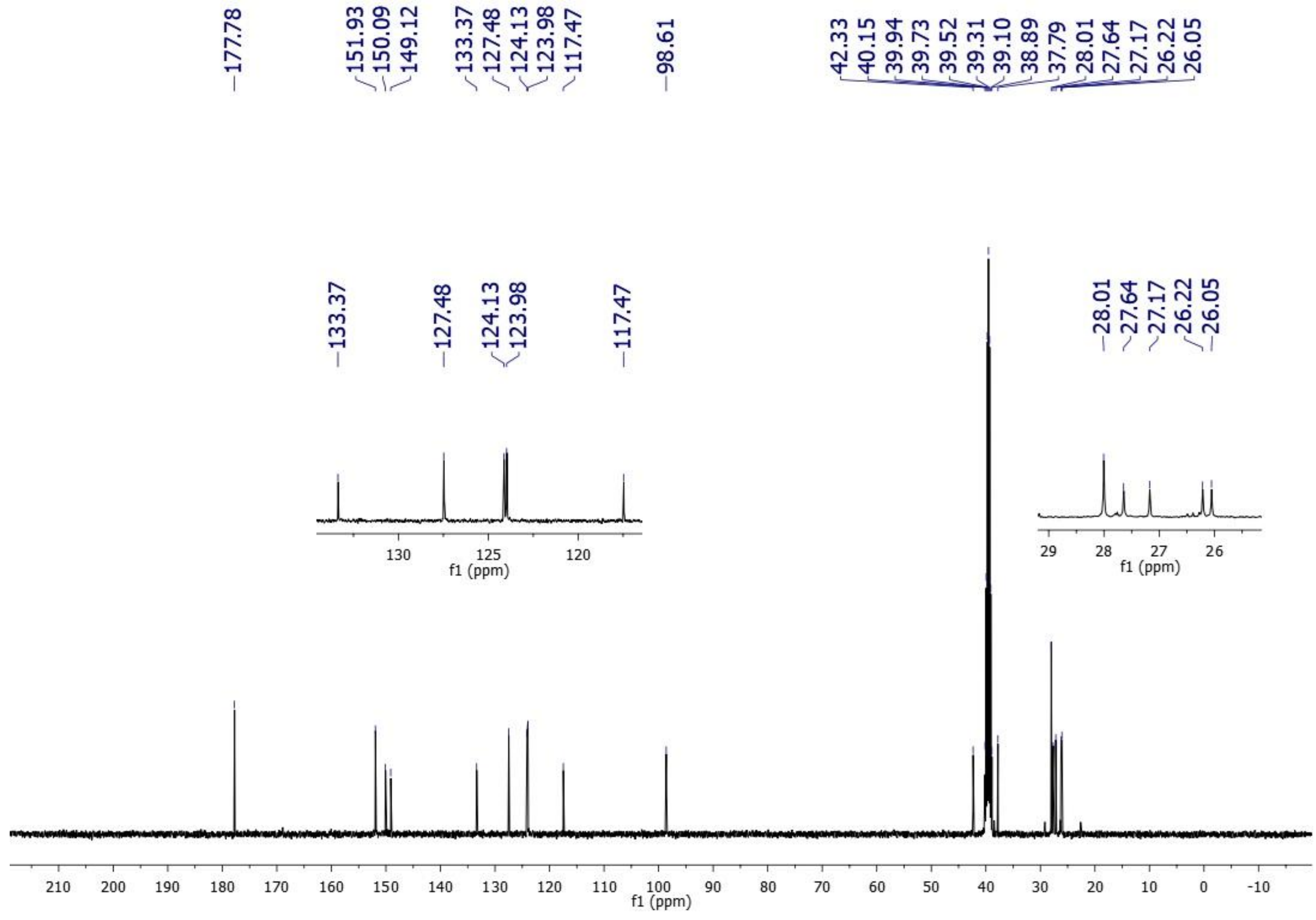

**Figure S17.** $^{13}C$ NMR spectrum (DMSO-$d_6$, 101 MHz) of compound **3d**.

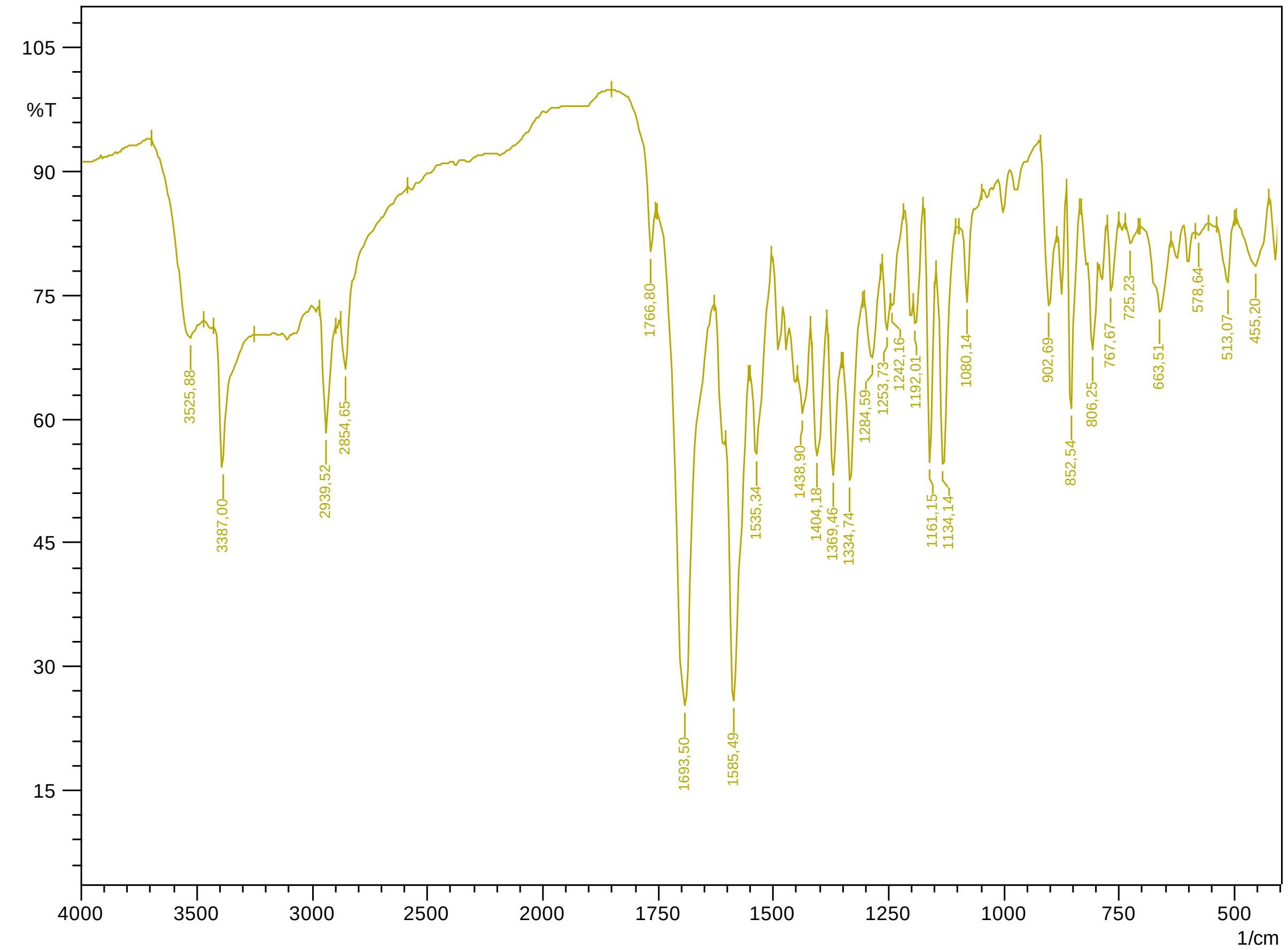

**Figure S18.** IR spectrum (KBr) of compound **3d**.

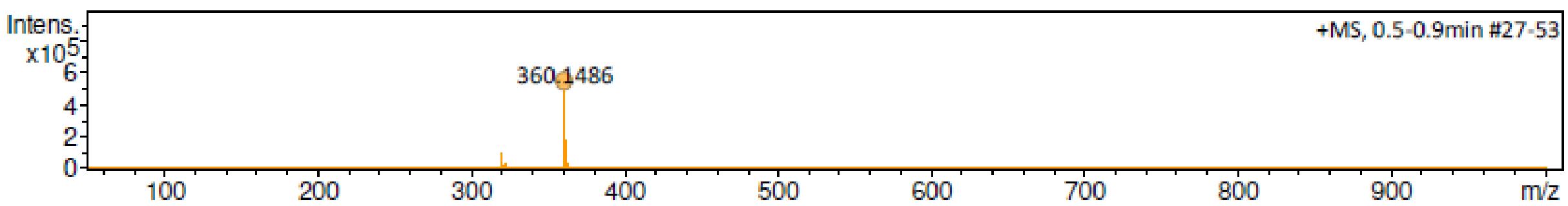

| Meas. m/z | # | Ion Formula | m/z | err [ppm] | mSigma | # mSigma | Score | rdb | e⁻ Conf | N-Rule |
|---|---|---|---|---|---|---|---|---|---|---|
| 360.1486 | 1 | C19H23ClN3O2 | 360.1473 | -3.4 | 1.7 | 1 | 100.00 | 9.5 | even | ok |

**Figure S19.** HRMS spectrum (ESI) of compound **3d**.

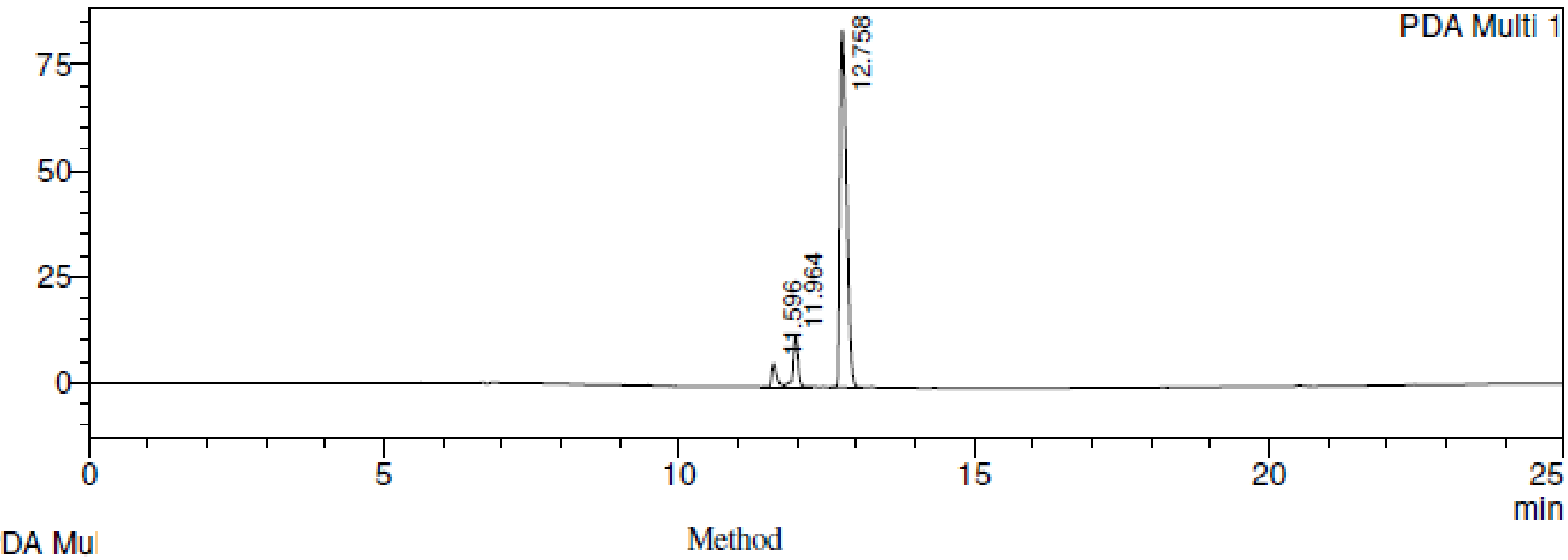


Method

<<Comment>>

<<LC Program>>

| Time | Unit | Command | Value |
|---|---|---|---|
| 0.01 | Pumps | B.Conc | 5 |
| 20.00 | Pumps | B.Conc | 100 |
| 25.00 | Pumps | B.Conc | 100 |
| 26.00 | Pumps | B.Conc | 5 |
| 46.00 | Controller | Stop | |

PeakTable

PDA Ch1 355nm 4nm

| Peak# | Ret. Time | Area | Area % |
|---|---|---|---|
| 1 | 11.596 | 32585 | 4.300 |
| 2 | 11.964 | 68023 | 8.976 |
| 3 | 12.758 | 657243 | 86.725 |
| Total | | 757851 | 100.000 |

Purity

Peak# : 3
Retention Time : 12.758
Compound Name :

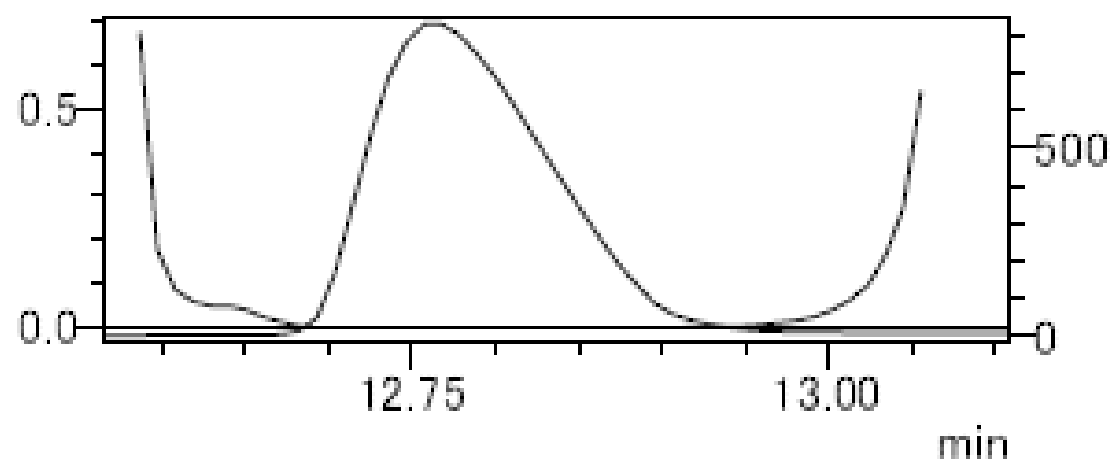


Impurity :Not Detected
Peak purity index : 1.000000
Single point threshold : 0.999971
Minimum peak purity index : 28

**Figure S20.** HPLC chromatogram of compound **3d**.

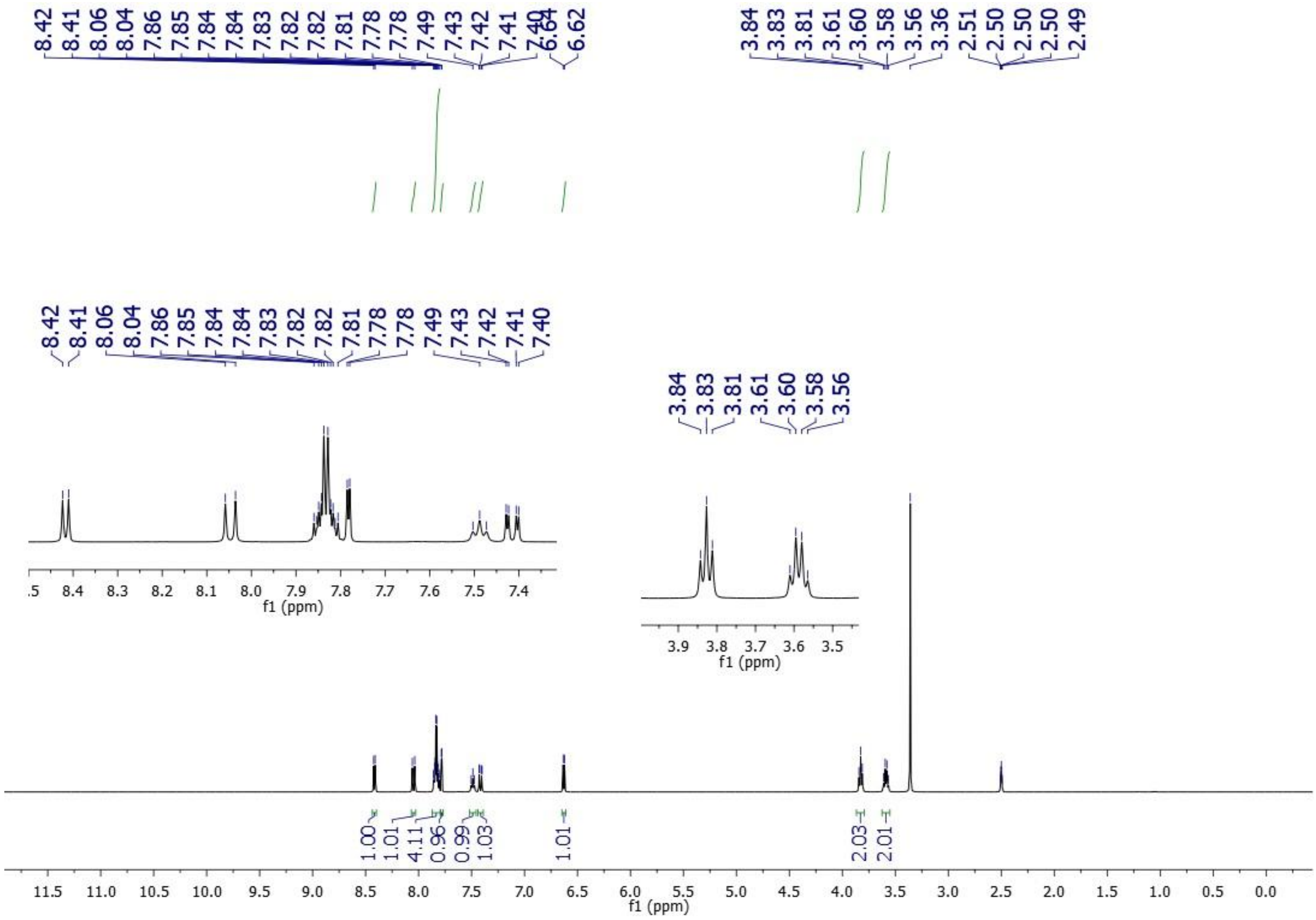

**Figure S21.** $^{1}$H NMR spectrum (DMSO-$d_6$, 400 MHz) of compound **5a**.

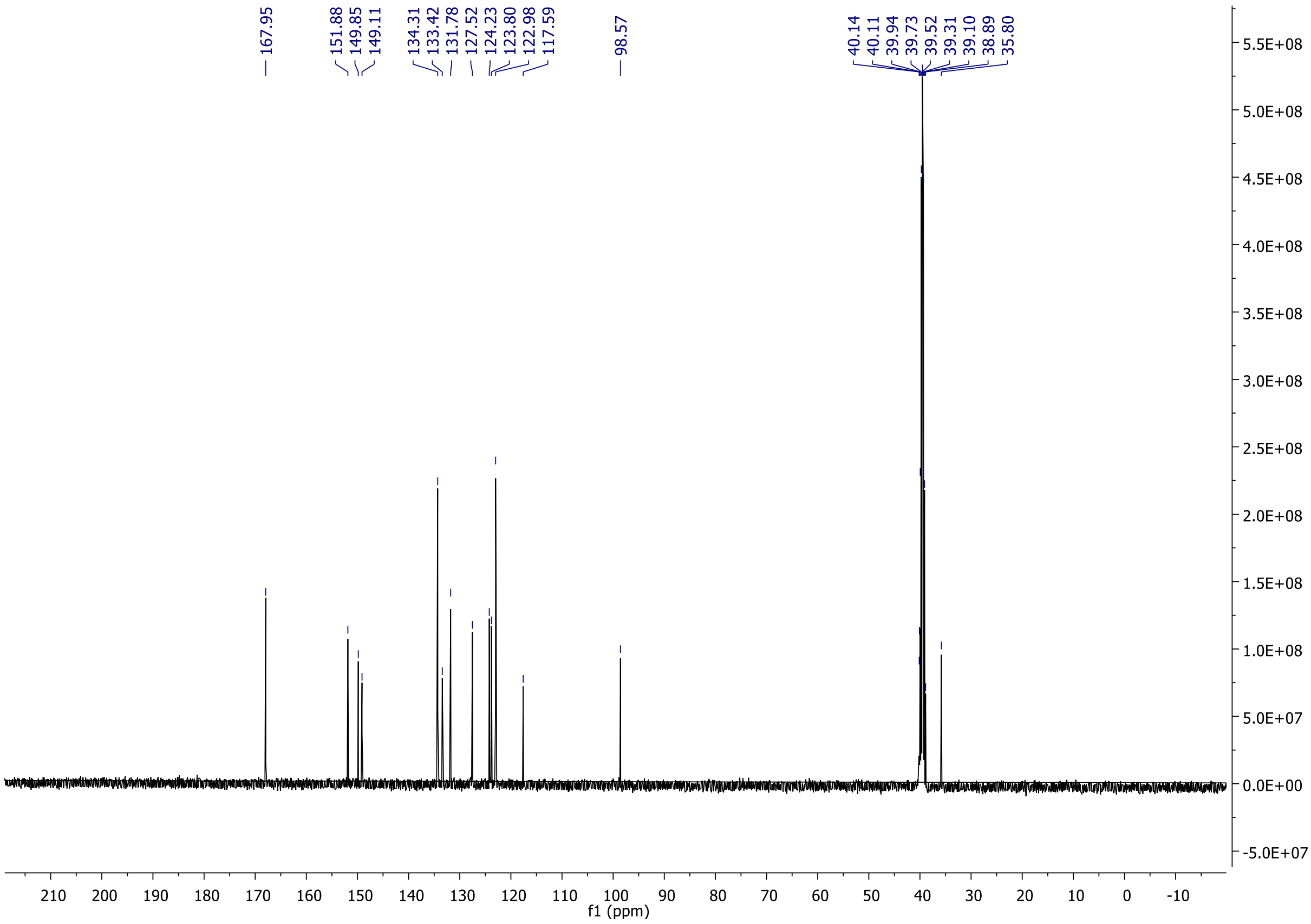

**Figure S22.** $^{13}$C NMR spectrum (DMSO-$d_6$, 101 MHz) of compound **5a**.

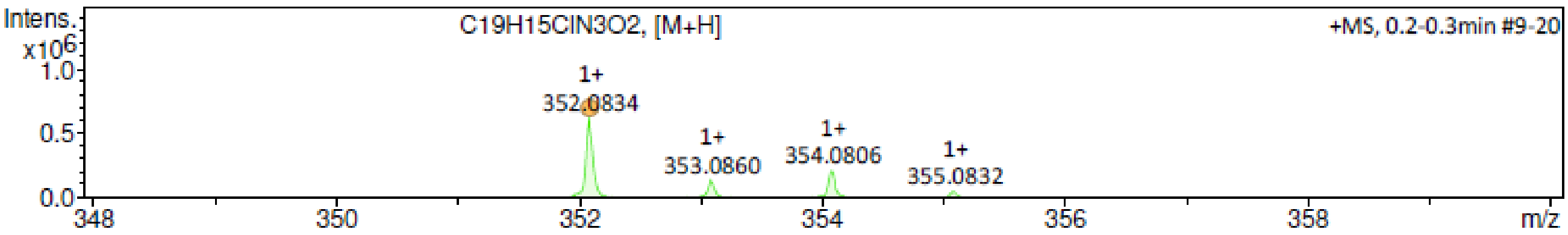


| Meas. m/z | # | Ion Formula | Score | m/z | err [mDa] | err [ppm] | mSigma | rdb | e⁻ Conf | N-Rule | Adduct |
|---|---|---|---|---|---|---|---|---|---|---|---|
| 352.083396 | 1 | C19H15ClN3O2 | 100.00 | 352.084731 | 1.3 | 3.8 | 5.6 | 13.5 | even | ok | M+H |

**Figure S23.** HRMS spectrum (ESI) of compound **5a**.

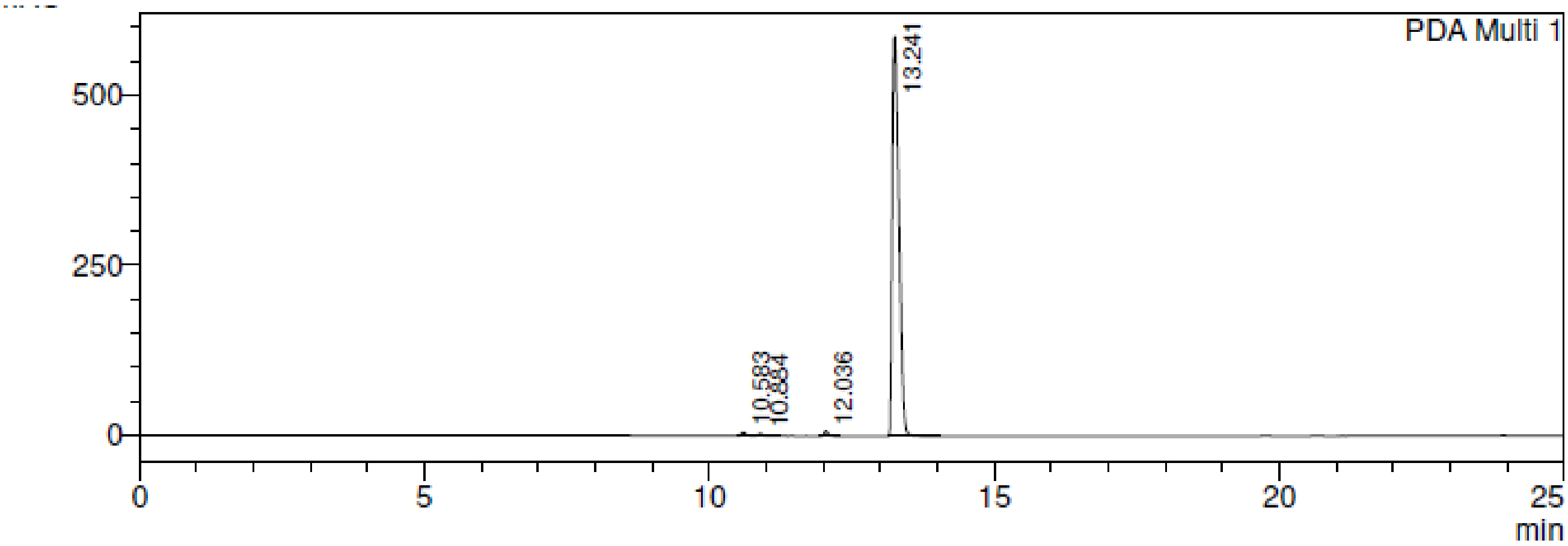


PDA Mul

Method

<<Comment>>
<<LC Program>>

| Time | Unit | Command | Value |
|---|---|---|---|
| 0.01 | Pumps | B.Conc | 5 |
| 20.00 | Pumps | B.Conc | 100 |
| 25.00 | Pumps | B.Conc | 100 |
| 26.00 | Pumps | B.Conc | 5 |
| 46.00 | Controller | Stop | |

PeakTable

PDA Ch1 343nm 4nm

| Peak# | Ret. Time | Area | Area % |
|---|---|---|---|
| 1 | 10.583 | 28036 | 0.572 |
| 2 | 10.884 | 17477 | 0.357 |
| 3 | 12.036 | 35540 | 0.725 |
| 4 | 13.241 | 4820874 | 98.347 |
| Total | | 4901927 | 100.000 |

Purity

Peak# : 4
Retention Time : 13.241
Compound Name :

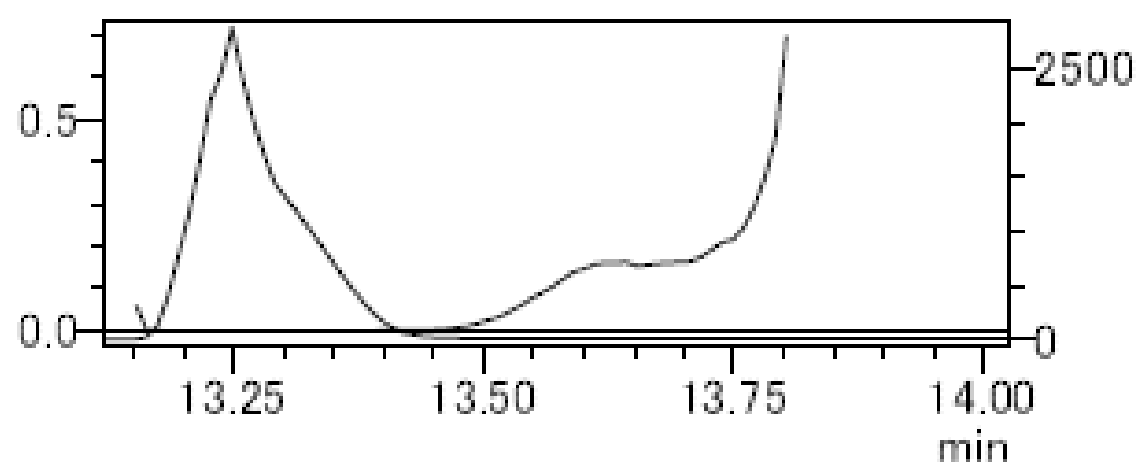


Impurity :Detected at 13.25 min
Peak purity index : 0.999688
Single point threshold : 0.999979
Minimum peak purity index : -290

**Figure S24.** HPLC chromatogram of compound **5a**.

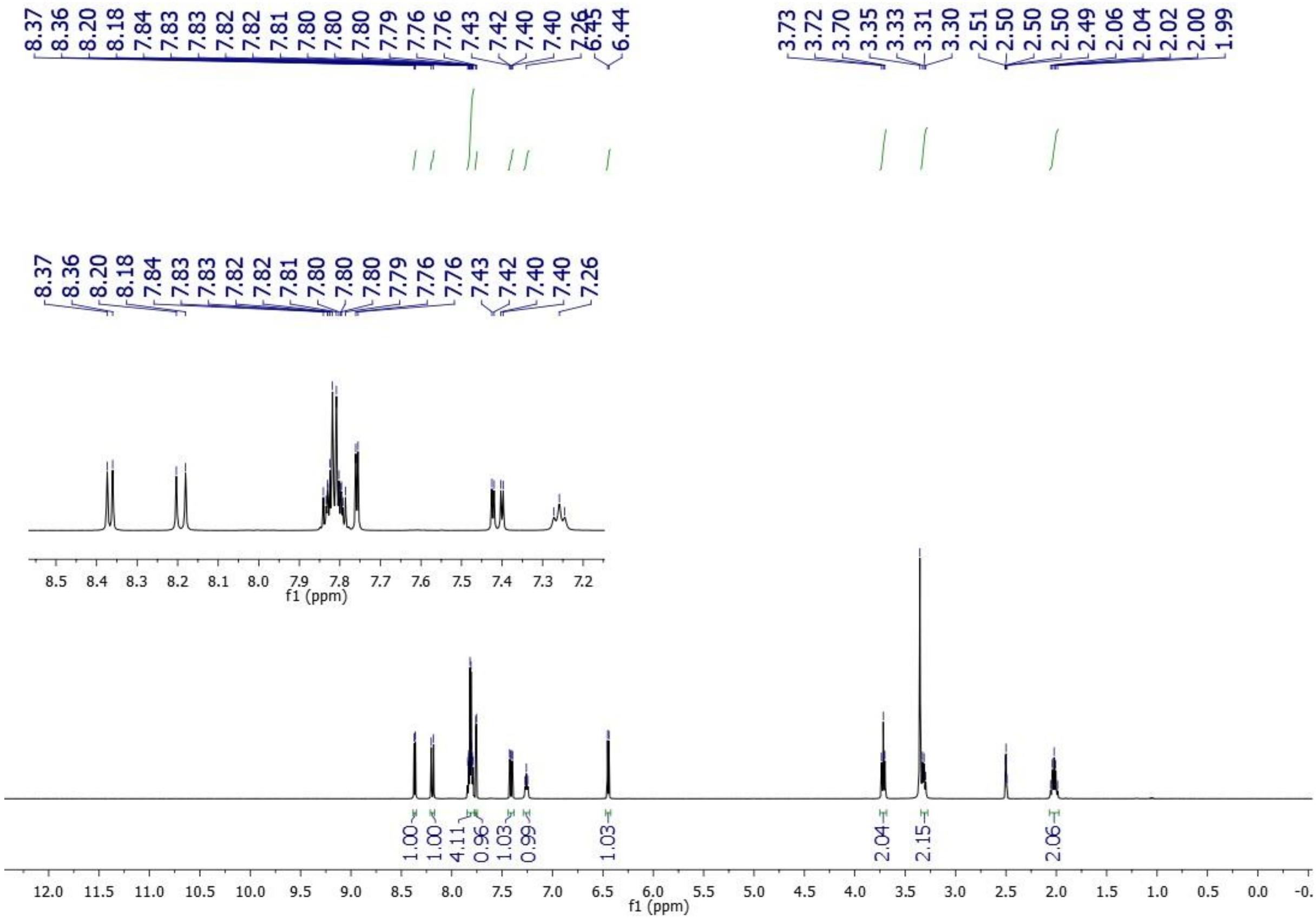

**Figure S25.** $^{1}H$ NMR spectrum (DMSO-$d_6$, 400 MHz) of compound **5b**.

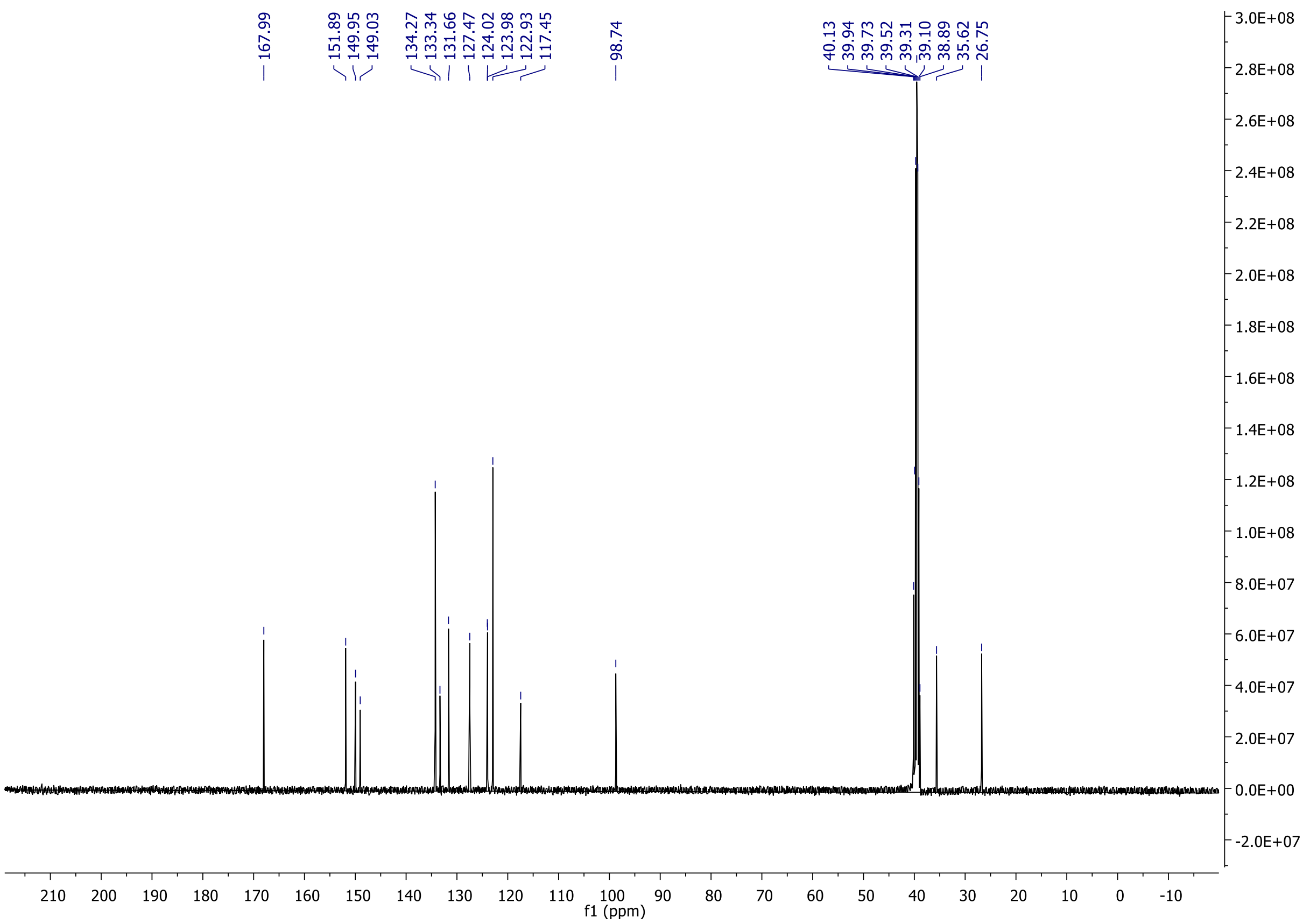

**Figure S26.** $^{13}C$ NMR spectrum (DMSO-$d_6$, 101 MHz) of compound **5b**.

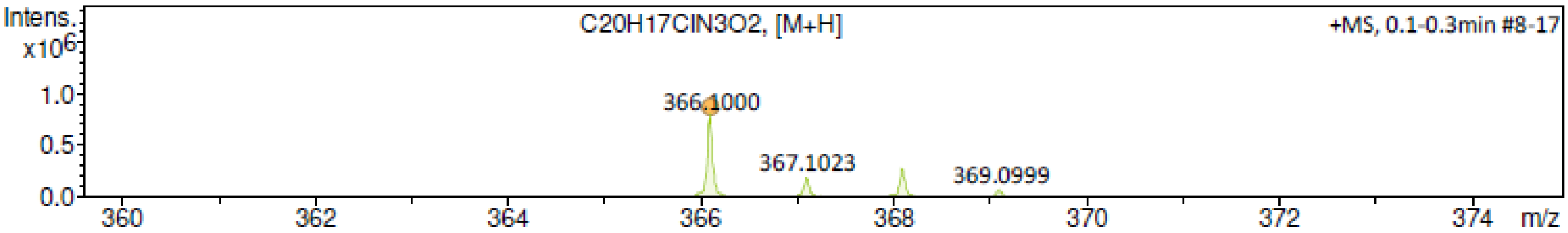


| Meas. m/z | # | Ion Formula | m/z | err [ppm] | mSigma | # mSigma | Score | rdb | e⁻ Conf | N-Rule |
|---|---|---|---|---|---|---|---|---|---|---|
| 366.1000 | 1 | C20H17ClN3O2 | 366.1004 | 1.2 | 4.5 | 1 | 100.00 | 13.5 | even | ok |

**Figure S27.** HRMS spectrum (ESI) of compound **5b**.

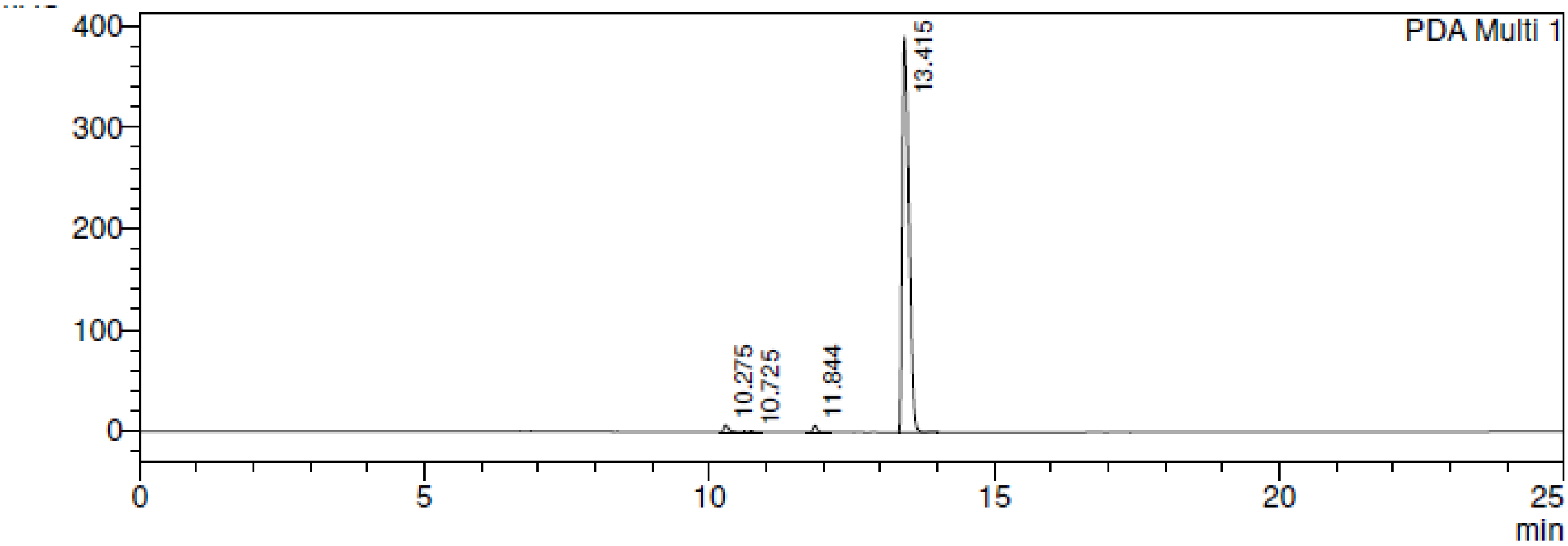


PDA Mul

Method

<<Comment>>

<<LC Program>>

| Time | Unit | Command | Value |
|---|---|---|---|
| 0.01 | Pumps | B.Conc | 5 |
| 20.00 | Pumps | B.Conc | 100 |
| 25.00 | Pumps | B.Conc | 100 |
| 26.00 | Pumps | B.Conc | 5 |
| 46.00 | Controller | Stop | |

PeakTable

PDA Ch1 343nm 4nm

| Peak# | Ret. Time | Area | Area % |
|---|---|---|---|
| 1 | 10.275 | 42245 | 1.291 |
| 2 | 10.725 | 7098 | 0.217 |
| 3 | 11.844 | 32858 | 1.004 |
| 4 | 13.415 | 3189191 | 97.487 |
| Total | | 3271392 | 100.000 |

Purity

Peak# : 4
Retention Time : 13.415
Compound Name :

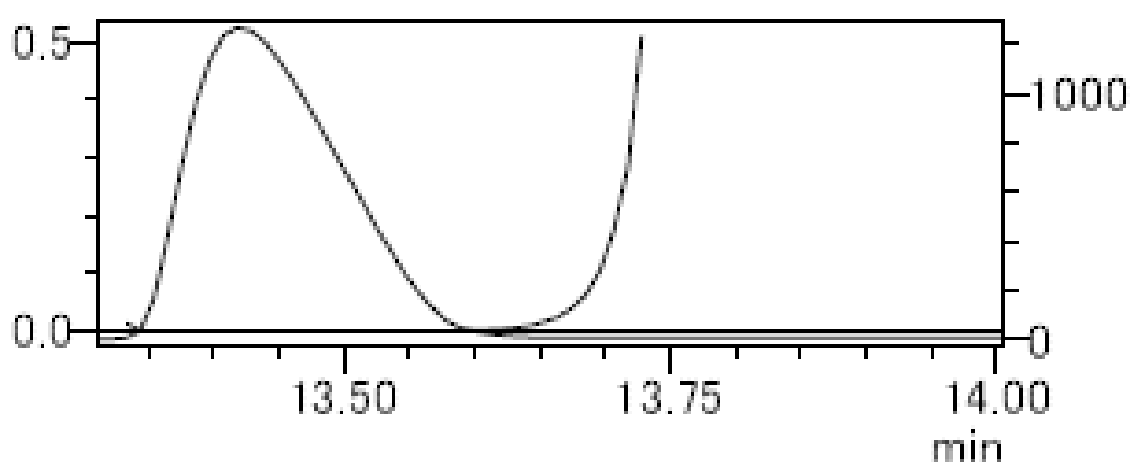


Impurity :Not Detected
Peak purity index : 1.000000
Single point threshold : 0.999961
Minimum peak purity index : 38

**Figure S28.** HPLC chromatogram of compound **5b**.

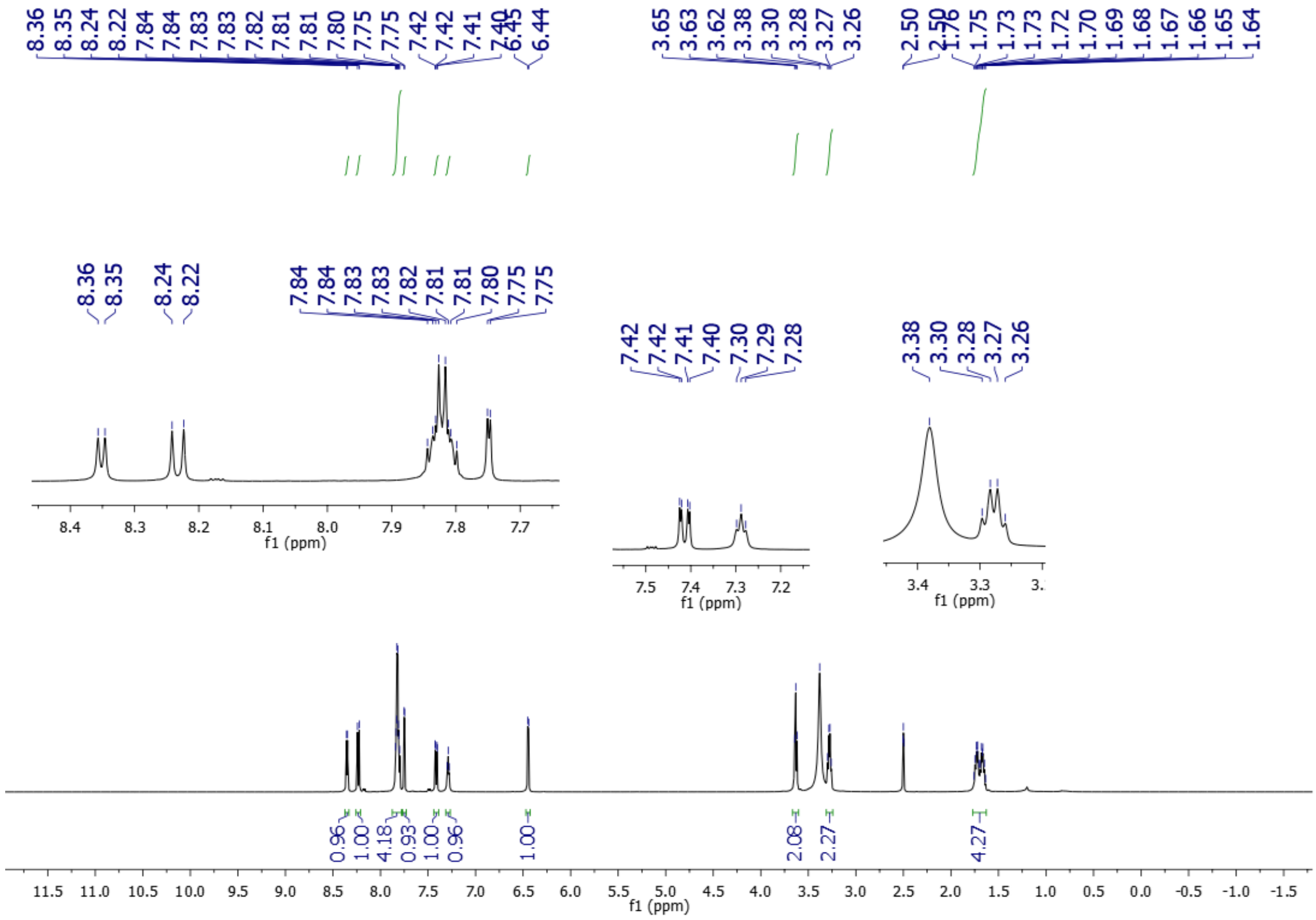


**Figure S29.** $^{1}H$ NMR spectrum (DMSO-$d_6$, 500 MHz) of compound **5c**.

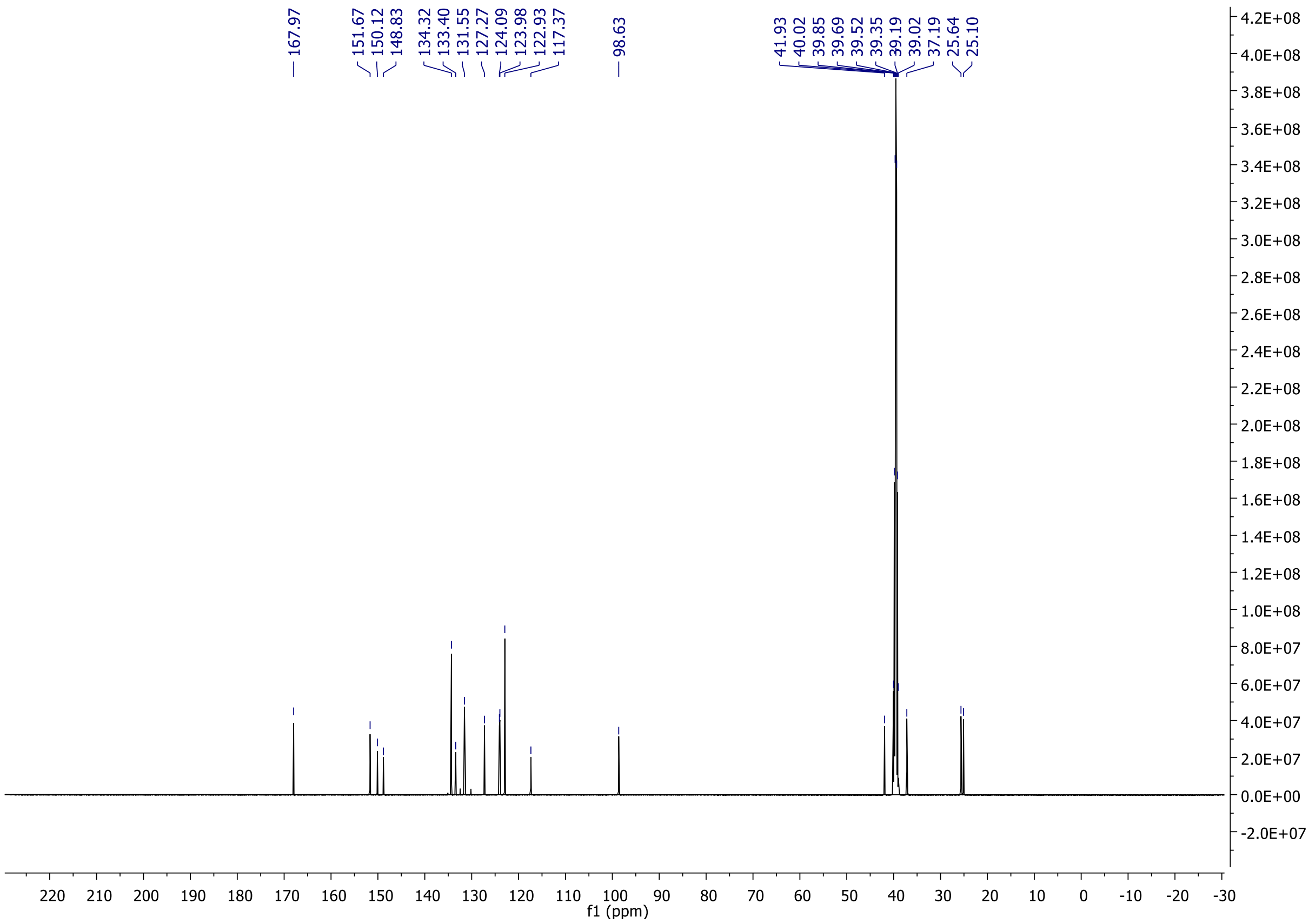


**Figure S30.** $^{13}C$ NMR spectrum (DMSO-$d_6$, 126 MHz) of compound **5c**.

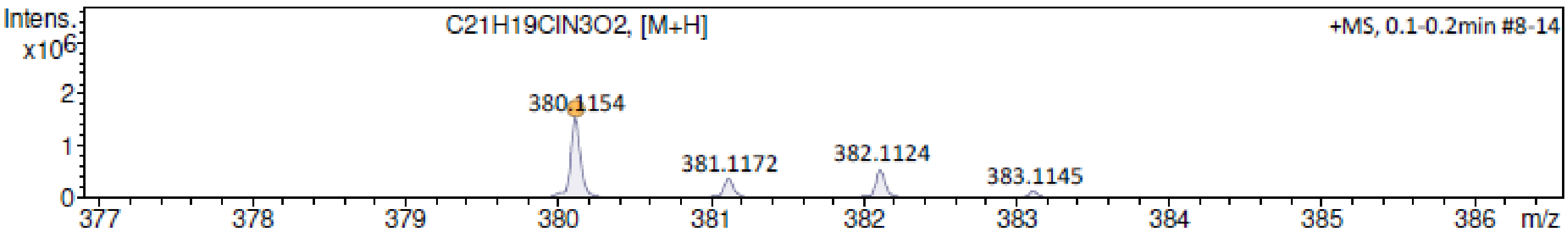


| Meas. m/z | # | Ion Formula | m/z | err [ppm] | mSigma | # mSigma | Score | rdb | e⁻ Conf | N-Rule |
|---|---|---|---|---|---|---|---|---|---|---|
| 380.1154 | 1 | C21H19ClN3O2 | 380.1160 | 1.7 | 1.7 | 1 | 100.00 | 13.5 | even | ok |

**Figure S31.** HRMS spectrum (ESI) of compound **5c**.

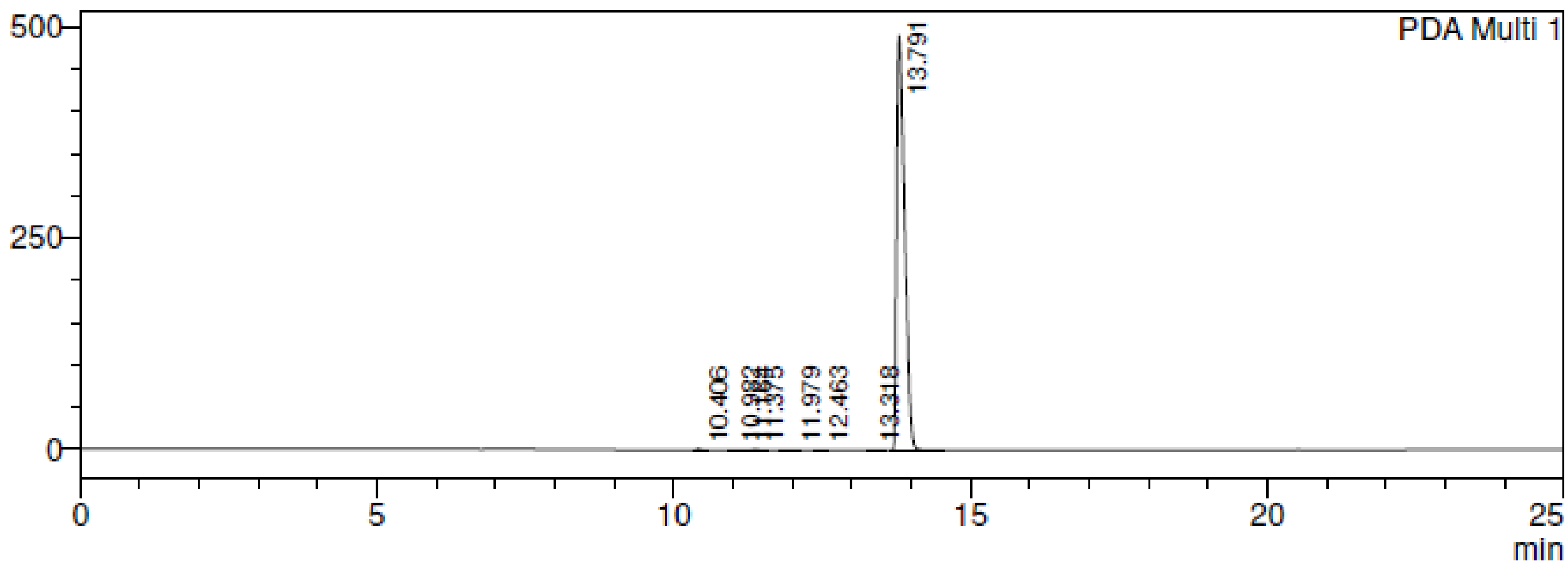


PDA Mul

Method

<<Comment>>
<<LC Program>>

| Time | Unit | Command | Value |
|---|---|---|---|
| 0.01 | Pumps | B.Conc | 5 |
| 20.00 | Pumps | B.Conc | 100 |
| 25.00 | Pumps | B.Conc | 100 |
| 26.00 | Pumps | B.Conc | 5 |
| 46.00 | Controller | Stop | |

PeakTable

PDA Ch1 343nm 4nm

| Peak# | Ret. Time | Area | Area % |
|---|---|---|---|
| 1 | 10.406 | 4613 | 0.099 |
| 2 | 10.982 | 1086 | 0.023 |
| 3 | 11.164 | 3225 | 0.070 |
| 4 | 11.375 | 5239 | 0.113 |
| 5 | 11.979 | 1403 | 0.030 |
| 6 | 12.463 | 1135 | 0.024 |
| 7 | 13.318 | 1753 | 0.038 |
| 8 | 13.791 | 4618302 | 99.602 |
| Total | | 4636756 | 100.000 |

Purity

Peak# : 8
Retention Time : 13.791
Compound Name :

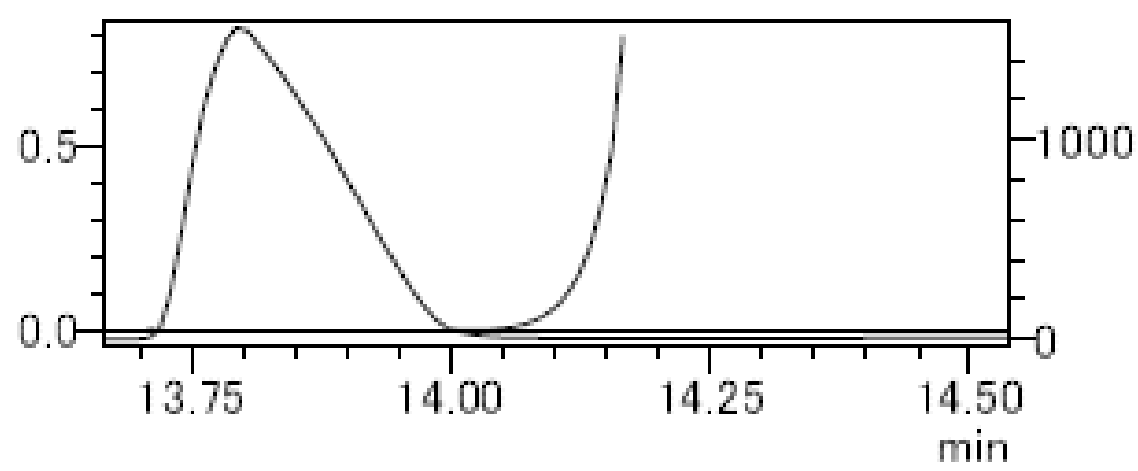


Impurity :Not Detected
Peak purity index : 0.999988
Single point threshold : 0.999968
Minimum peak purity index : 20

**Figure S32.** HPLC chromatogram of compound **5c**.

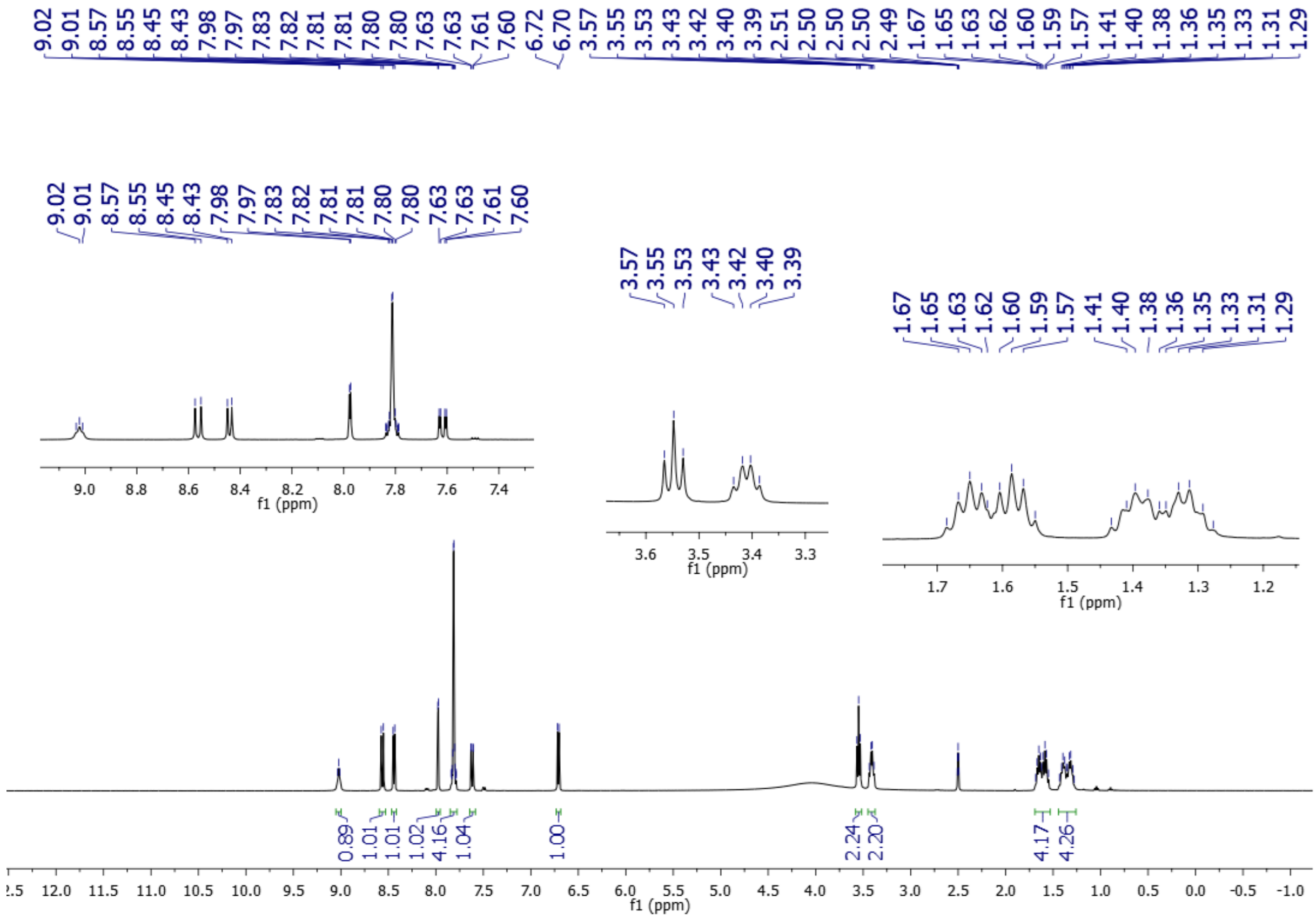

**Figure S33.** $^{1}$H NMR spectrum (DMSO-$d_6$, 400 MHz) of compound **5d**.

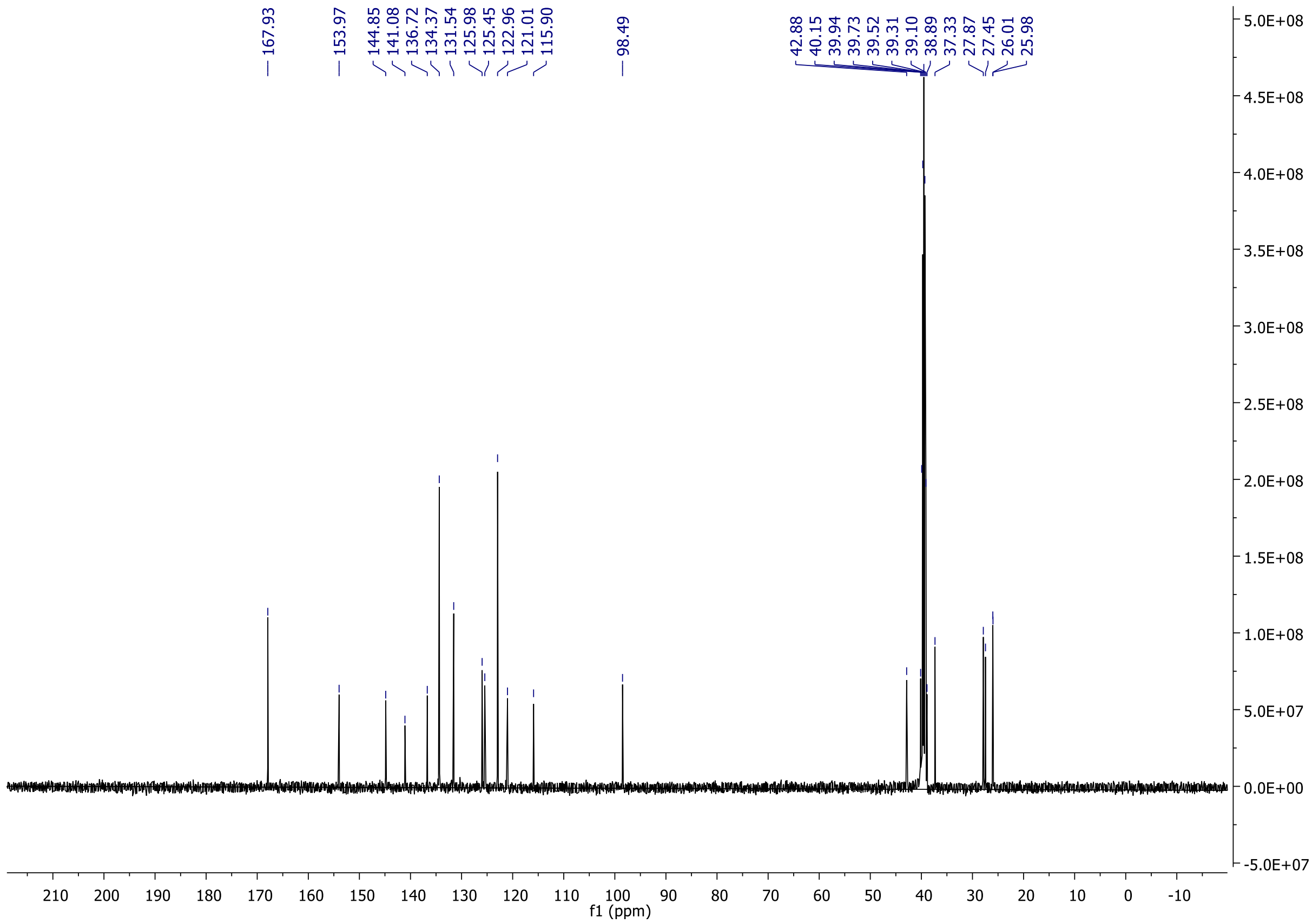

**Figure S34.** $^{13}$C NMR spectrum (DMSO-$d_6$, 101 MHz) of compound **5d**.

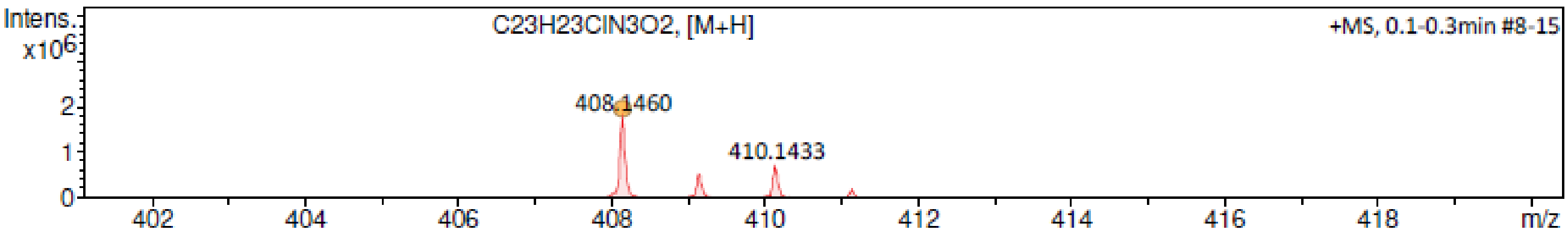


| Meas. m/z | # | Ion Formula | m/z | err [ppm] | mSigma | # mSigma | Score | rdb | e⁻ Conf | N-Rule |
|---|---|---|---|---|---|---|---|---|---|---|
| 408.1460 | 1 | C23H23ClN3O2 | 408.1473 | 3.4 | 14.8 | 1 | 100.00 | 13.5 | even | ok |

**Figure S35.** HRMS spectrum (ESI) of compound **5d**.

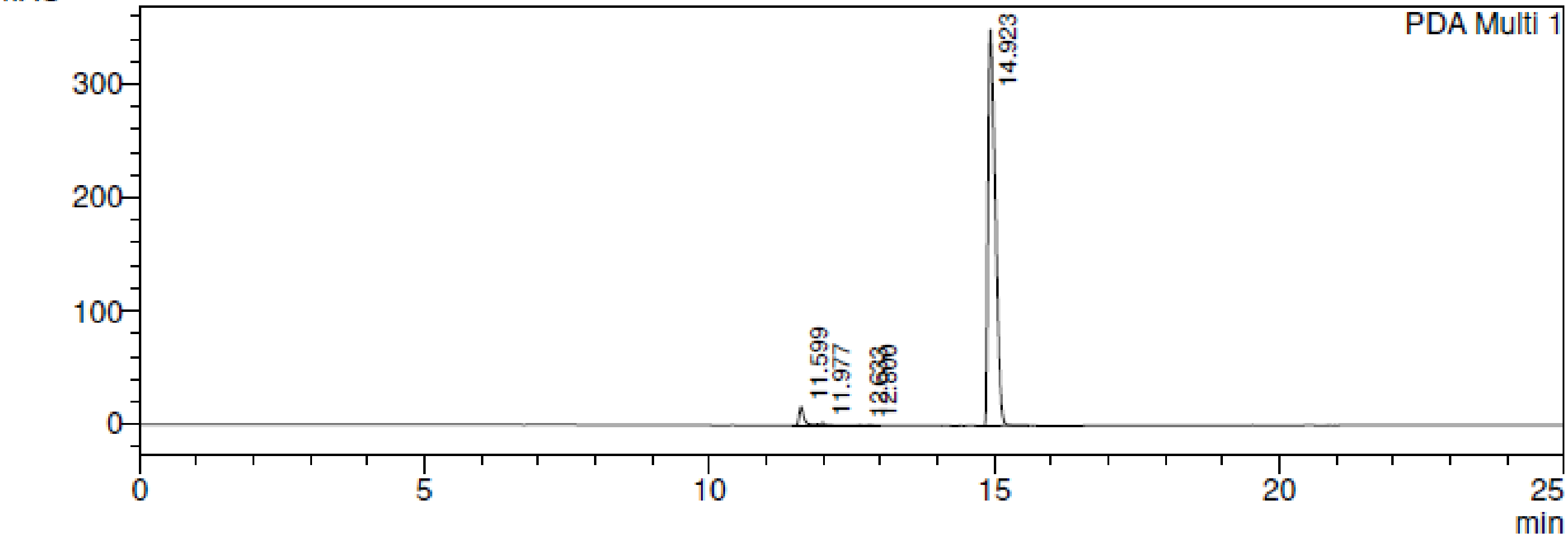


PDA Mul

Method

<<Comment>>

<<LC Program>>

| Time | Unit | Command | Value |
|---|---|---|---|
| 0.01 | Pumps | B.Conc | 5 |
| 20.00 | Pumps | B.Conc | 100 |
| 25.00 | Pumps | B.Conc | 100 |
| 26.00 | Pumps | B.Conc | 5 |
| 46.00 | Controller | Stop | |

PeakTable

PDA Ch1 343nm 4nm

| Peak# | Ret. Time | Area | Area % |
|---|---|---|---|
| 1 | 11.599 | 98518 | 3.028 |
| 2 | 11.977 | 18070 | 0.555 |
| 3 | 12.633 | 1647 | 0.051 |
| 4 | 12.800 | 2999 | 0.092 |
| 5 | 14.923 | 3132162 | 96.274 |
| Total | | 3253395 | 100.000 |

Purity

Peak# : 5
Retention Time : 14.923
Compound Name :

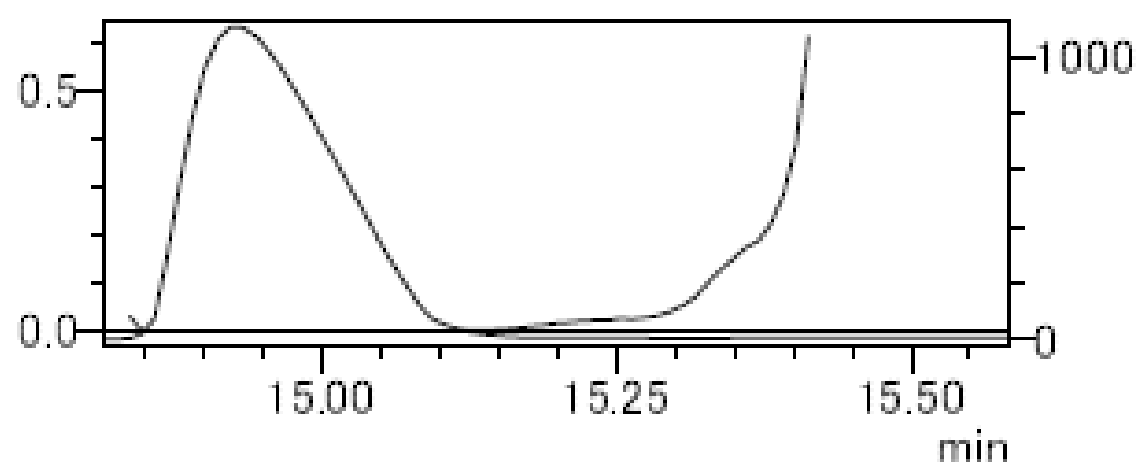


Impurity :Not Detected
Peak purity index : 0.999999
Single point threshold : 0.999960
Minimum peak purity index : 39

**Figure S36.** HPLC chromatogram of compound **5d**.

**Table S1.** Estimated 50% lethal concentration ($LC_{50}$) of compounds **2d**, **3a–c**, and ciprofloxacin hydrochloride on *Artemia salina* larvae after 24 h.

| Compound | Graphical |
|---|---|
| **2d** | 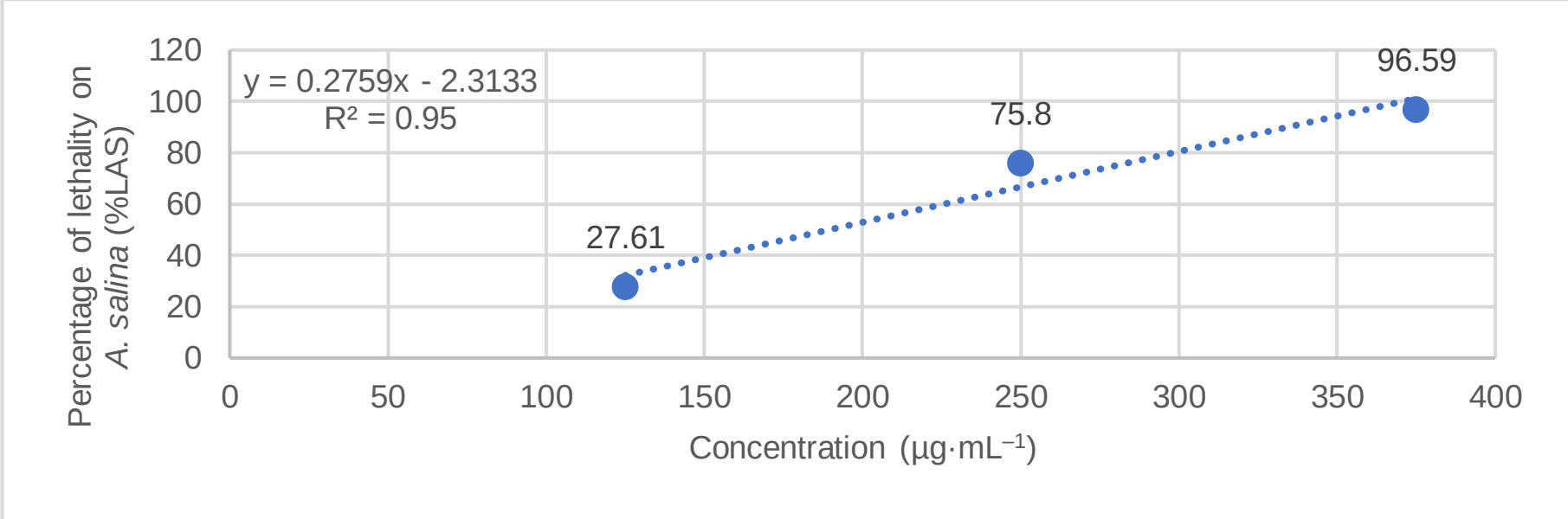  |
| **3a** | 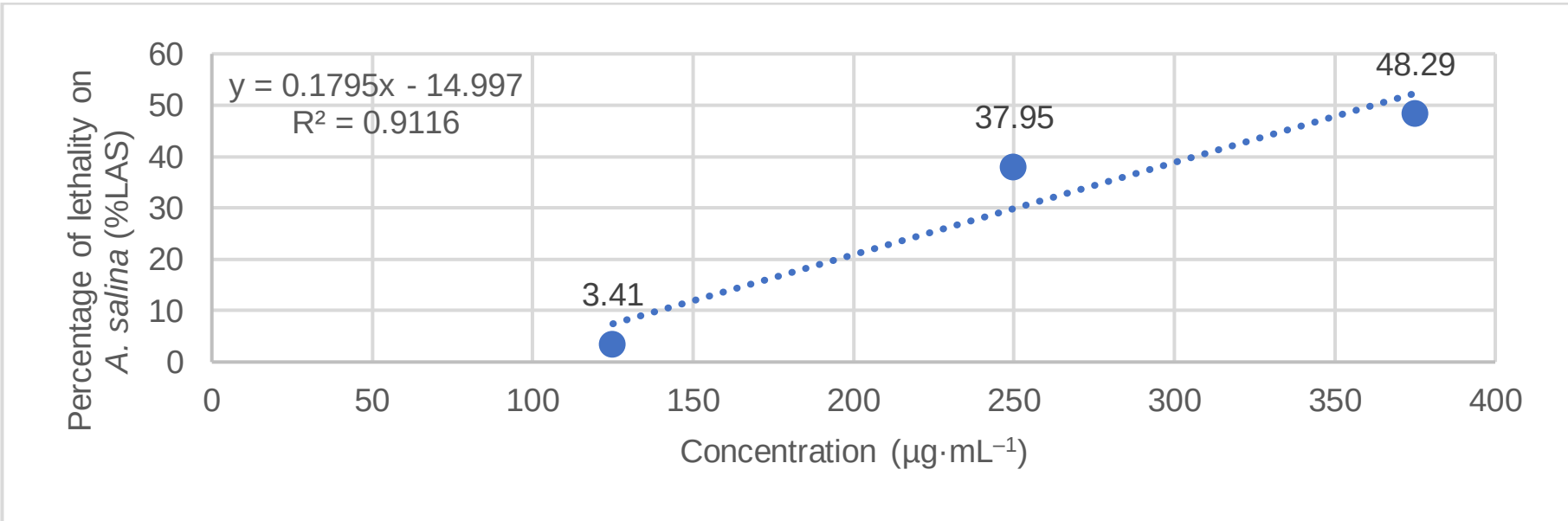  |
| **3b** | 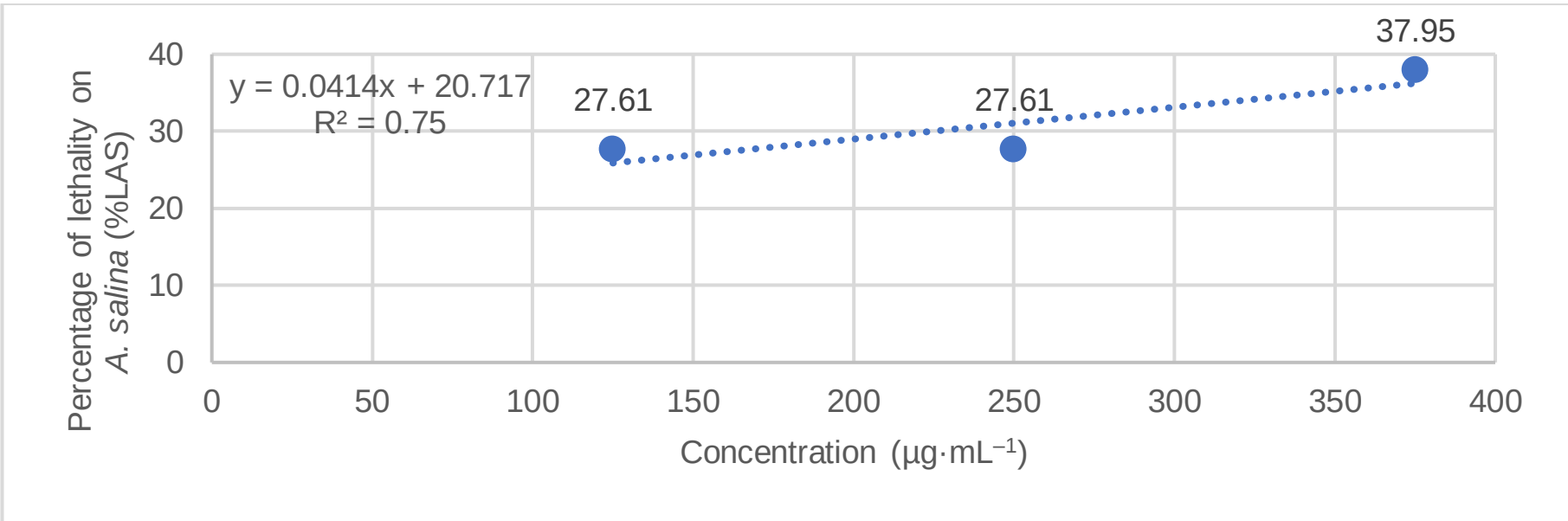  |
| **3c** | 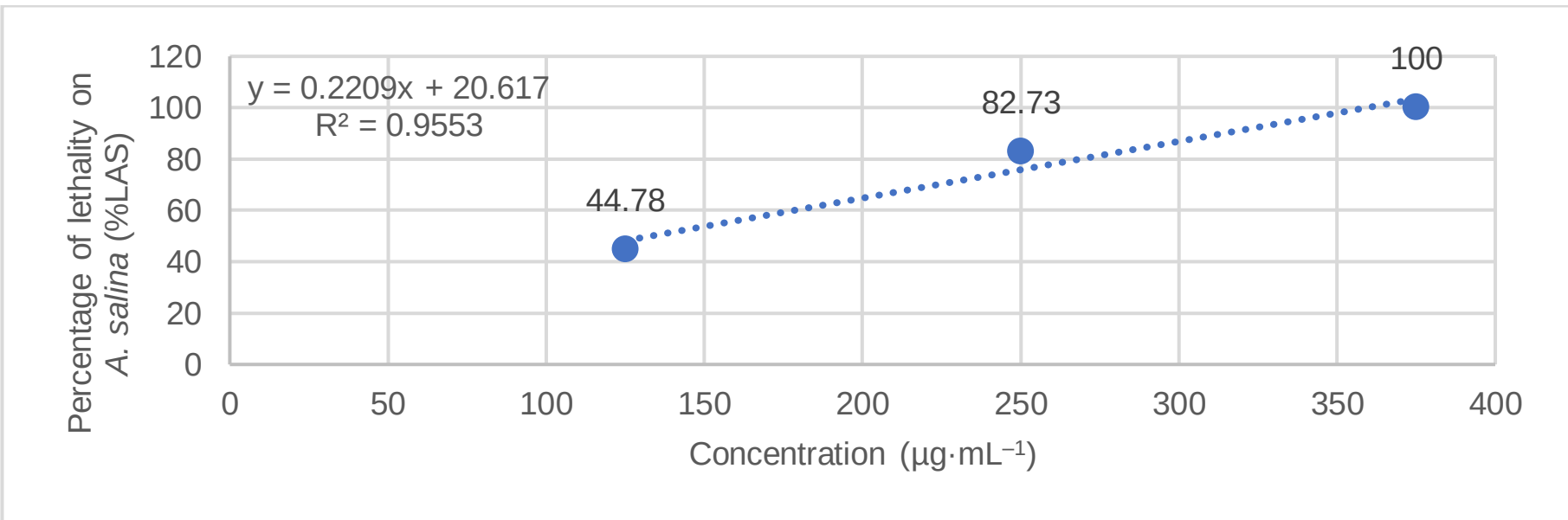  |
| Ciprofloxacin hydrochloride | 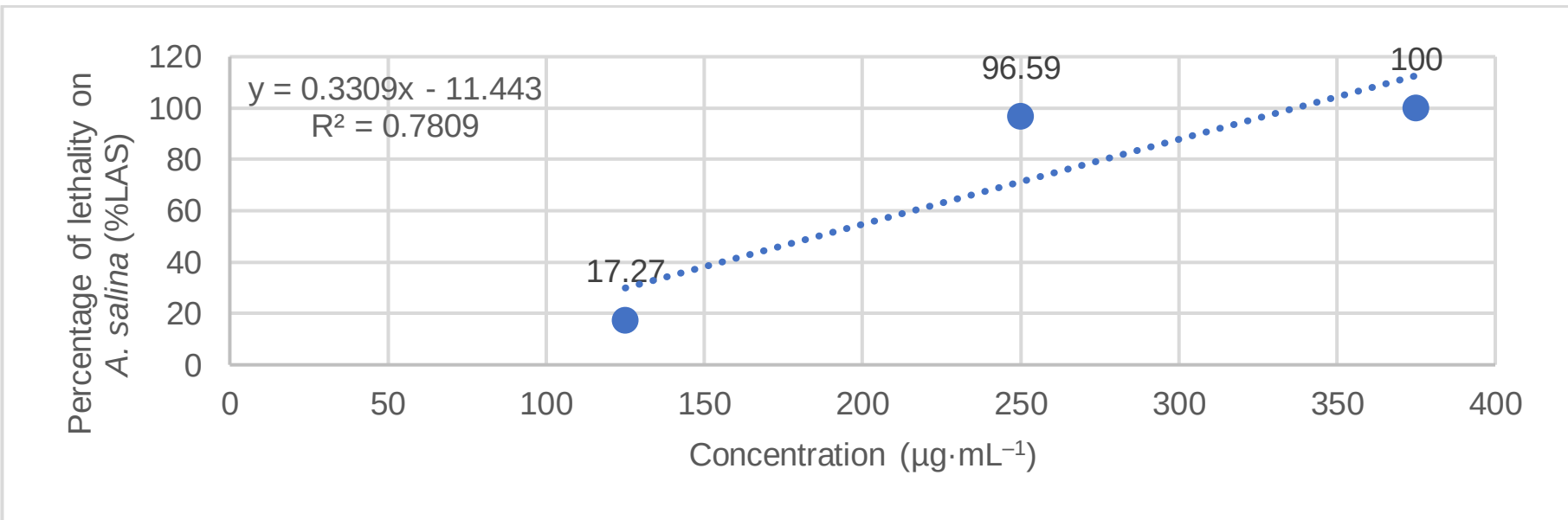  |

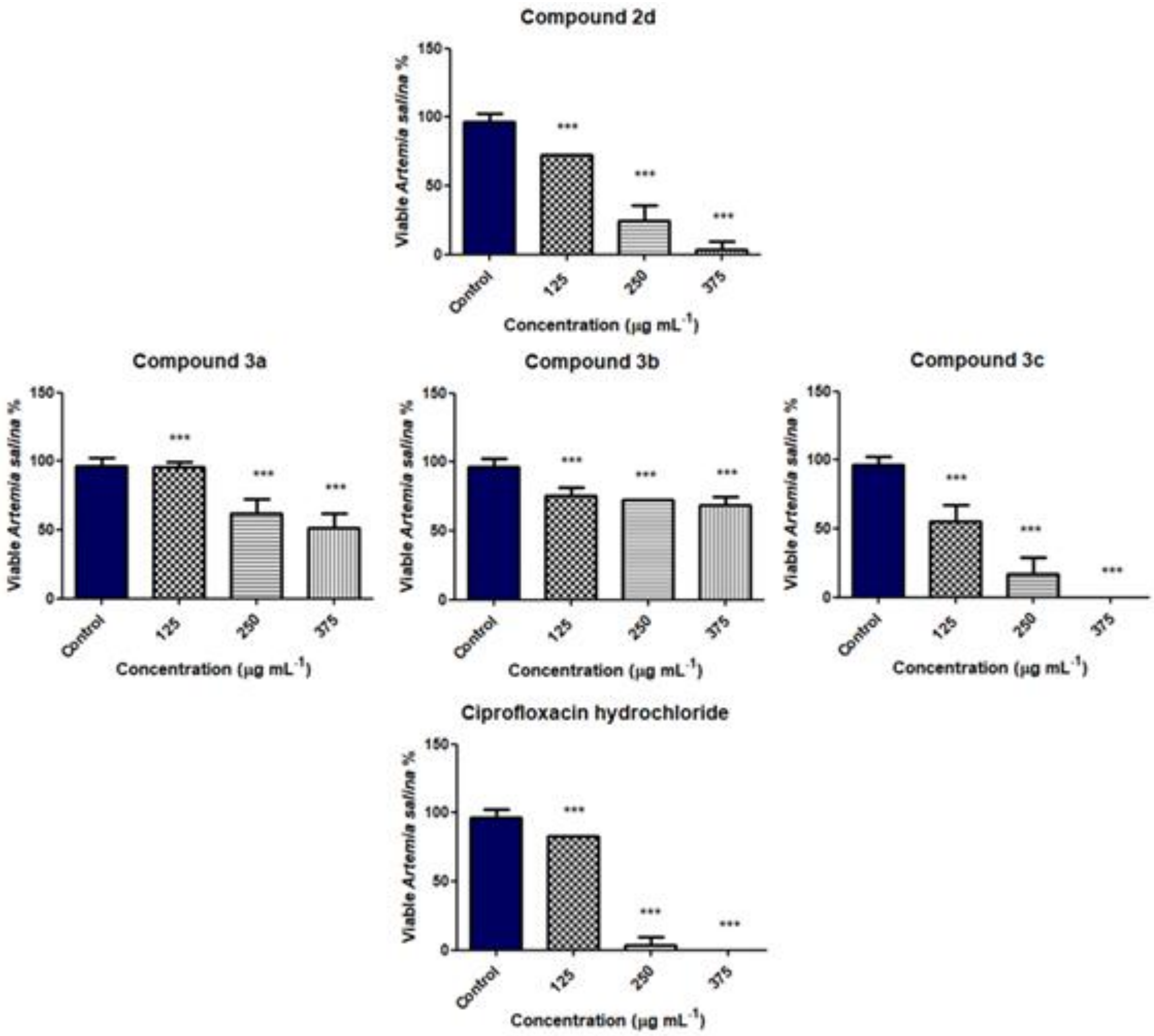


**Figure S37.** Average percentages of alive *A. salina* nauplii at different concentrations of compounds **2d**, **3a–c**, and ciprofloxacin hydrochloride after 24 h. Differences were considered statistically significant when P ≤ 0.0007*** by the one-way analysis of variance and Bonferroni's Multiple Comparison Test.
